# Chemical Fingerprinting, Isolation and Characterization of Polyphenol Compounds from *Heliotropium taltalense* (Phil.) I.M. Johnst and Its Endothelium-Dependent Vascular Relaxation Effect in Rat Aorta

**DOI:** 10.3390/molecules25143105

**Published:** 2020-07-08

**Authors:** Ruth E. Barrientos, Mario J. Simirgiotis, Javier Palacios, Adrián Paredes, Jorge Bórquez, Alejandra Bravo, Fredi Cifuentes

**Affiliations:** 1 Instituto de Farmacia, Facultad de Ciencias, Universidad Austral de Chile, Valdivia 5090000, Chile; ruth.barrientos@alumnos.uach.cl; 2 Laboratorio de Bioquímica Aplicada, Facultad de Ciencias de la Salud, Universidad Arturo Prat, Iquique 1110939, Chile; 3 Departamento de Química, Facultad de Ciencias Básicas, Universidad de Antofagasta, Casilla 170, Antofagasta 1240000, Chile; adrian.paredes@uantof.cl (A.P.); jorge.borquez@uantof.cl (J.B.); 4 Laboratorio de Fisiología Experimental (EPhyL), Instituto Antofagasta (IA), Universidad de Antofagasta, Antofagasta 1240000, Chile; alejandrabravo.bq@gmail.com (A.B.); fredi.cifuentes@uantof.cl (F.C.)

**Keywords:** UHPLC Q-Orbitrap HR-MS/MS, NMR, Chilean plants, endemic species, flavonoids, vasodilation, rat aorta

## Abstract

*Heliotropium taltalense* is an endemic species of the northern coast of Chile and is used as folk medicine. The polyphenolic composition of the methanolic and aqueous extract of the endemic Chilean species was investigated using Ultrahigh-Performance Liquid Chromatography, Heated Electrospray Ionization and Mass Spectrometry (UHPLC-Orbitrap-HESI-MS). Fifty-three compounds were detected, mainly derivatives of benzoic acid, flavonoids, and some phenolic acids. Furthermore, five major compounds were isolated by column chromatography from the extract, including four flavonoids and one geranyl benzoic acid derivative, which showed vascular relaxation and were in part responsible for the activity of the extracts. Since aqueous extract of *H. taltalense* (83% ± 9%, 100 μg/mL) produced vascular relaxation through an endothelium-dependent mechanism in rat aorta, and the compounds rhamnocitrin (89% ± 7%; 10^−4^ M) and sakuranetin (80% ± 6%; 10^−4^ M) also caused vascular relaxation similar to the extracts of *H. taltalense*, these pure compounds are, to some extent, responsible for the vascular relaxation.

## 1. Introduction

**Figure 1:**
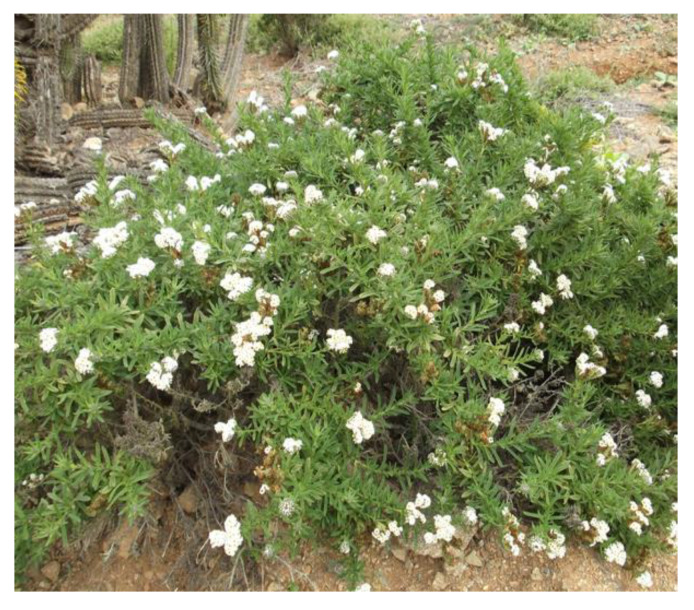
Picture of *Heliotropium taltalense* collected in Paposo Valley, Atacama Desert, in April 2016.

The endemic species *Heliotropium taltalense* (Phil.) I.M. Johnst. (Heliotropiaceae) called Monte negro in Chile has been used since pre-Hispanic times as an anti-inflammatory and to treat bone bruises [1]. *H. taltalense* is a species with white and yellow flowers (Figure 1), which grows in the Chilean coastal area of Paposo valley at an altitude of 0–500 m above the sea level.

Currently, prevalence rates of hypertension are on the rise in developing countries [2]. Thus, it is very convenient to search for new extracts or metabolites, in the folkloric use of medicinal plants, for the control of blood pressure. Several vascular pathologies find their cause on inflammatory and oxidative mechanisms [3]. Therefore, the anti-inflammatory effect described for this plant led us to think that *H. taltalense* extracts or its isolated bioactive compounds can be useful for the control of the vascular response, and then, the control of blood pressure.

The resin of this species is a producer of several interesting benzofuran derivatives and benzoic acid geranyl derivatives with several tested biological properties [4,5,6]. So far, only the exudates of this plant have been investigated [4,7]. HPLC hyphenated with mass spectrometry is today a key tool for the fast small-molecule analysis of herbal samples [8,9] and the high resolution Q-Orbitrap technology have demonstrated to be fast and accurate [10] and very reliable for metabolite profiling including antibiotics [10], vegetables [11], fruits [12] and plant resins [7] among others.

Pharmacological studies on rat aorta are useful tools for the evaluation of vascular endothelium and smooth muscle in vitro [13]. To assess vascular relaxation of chemical substances, the rat aorta should first be pre-contracted with available agonists (e.g., KCl or phenylephrine), and then, the vascular contractile response must reach a plateau before adding the vascular relaxation substances [14]. Flavonoids possess a C6-C3-C6 basic skeleton, with different OH substitution patterns, which can be glycosylated or methylated and with several reported bioactivities such as scavenging of harmful radicals [15], anticancer [16], and anti-inflammatory [17]. On the other hand, several flavonoids of different subclasses including flavanones, flavones, flavonols, and flavanes were reported to show relaxant effects dependent on the concentration in rat aorta pre-contracted with KCl or phenylephrine (PE) [18]. Dietary flavones indeed protect against cardiovascular disease [19], besides, rat aortas from flavonoid-treated animals showed good endothelium-dependent relaxations to acetylcholine, to a similar extent as those pretreated with the angiotensin-converting enzyme inhibitor, captopril [20].

In the present work we have tested the extract and five metabolites isolated (four flavones and one benzoic acid derivative) for vascular responses in a rat model. Besides, we discuss the tentative identification of all phenolic compounds in the extracts by UHPLC Q-Orbitrap High-Resolution-MS/MS, plus the bioactivities of the five major compounds isolated. We also evaluated the antioxidant activity of the methanolic and aqueous extracts of *H. taltalense*. To the best of our knowledge, no vascular responses have been studied using pure compounds isolated and extracts of *H. taltalense*.

## 2. Results and Discussion

### 2.1. Identification of Phenolic Compounds in H. taltalense Methanolic and Aqueous Extracts

**Figure 2:**
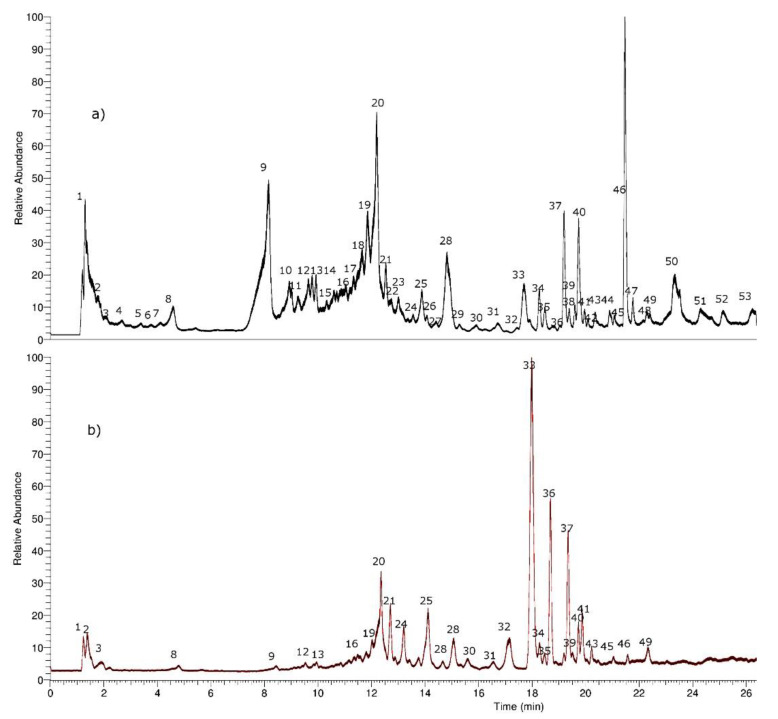
Total ion current (TIC) chromatograms of *H. taltalense*: (**a**) methanolic extract and (**b**) aqueous extract.

The first step for the characterization of the compounds in the UHPLC fingerprints was their classification based on their UV spectra followed by the accurate determination of the molecular weight in the negative mode. In the negative ionization mode (ESI^−^) spectrum, the most intense peak usually corresponds to the deprotonated molecular ion [M − H]^−^. Compounds were numbered in the total ion current (TIC) chromatograms by their order of elution (Figure 2). Fifty-three compounds were identified or tentatively identified by means of high-resolution MS and Photo-Diode Array (PDA) detection. All fifty-three compounds were detected in the methanolic extract, but twelve of those peaks were not detected in the aqueous extract (peaks 5, 6, 7, 10, 11, 14, 26, 38, and 50–53) however, peaks 33, 36, and 37 were more abundant (by its relative intensity) in the aqueous extract, so probably these compounds present some additional enhanced vascular activity (Supplementary Material Table S1, and Figure S23a–j).

#### 2.1.1. Phenolic Acids

Peak 13 with a pseudo-molecular ion at *m*/*z* 359.07703 and characteristic daughter ions at *m*/*z* 161.02396 (C_9_H_5_O_3_^−^); 179.03428 (C_9_H_7_O_4_^−^ caffeic acid) was identified as rosmarinic acid (C_27_H_29_O_15_^−^), peak 8 as caffeic acid (C_9_H_7_O_3_^−^), and peak 15 as its methyl derivative ferulic acid (C_10_H_9_O_4_^−^). Peak 17 (ion at *m*/*z* 137.02365) was identified as 4-*O*-methoxyferulic acid (C_7_H_5_O_3_^−^) and peak 25 (ion at *m*/*z* 207.06580) as 4-*O*-methyl ferulic acid (C_11_H_11_O_4_^−^), while peaks 3, 4, and 5 were identified as 2,4,5-trihydroxybenzoic acid, 4-hydroxy-3,5-dimethoxybenzoic acid, and 2,4-dihydroxybenzoic acid, (C_6_H_5_O_5_^−^, C_9_H_9_O_5_^−^, and C_7_H_5_O_4_^−^) respectively.

#### 2.1.2. Organic Saturated Acids

Peaks 1 and 2 were identified as malic and citric acids (C_4_H_5_O_5_^−^ and C_6_H_7_O_7_^−^), respectively. Peak 6 with a pseudo-molecular ion at *m*/*z* 161.08122 was identified as dihydroxyheptanoic acid (C_7_H_13_O_4_^−^) and peak 7 as its derivative, dihydroxyheptanoic acid glucoside (C_13_H_23_O_9_^−^).

#### 2.1.3. Oxylipins

Peak 11 was identified as the oxylipin glucoside derivative [21] tetrahydroxy-tetradecadienoic acid-*O*-glucoside (C_20_H_33_O_11_^−^).

#### 2.1.4. Benzoic Acid Derivatives

Several compounds were already reported by us, including 3-*H*-spiro 1-benzofuran-2,1′-cyclohexane derivatives and geranyl benzoic derivatives, which were present in the resin exudate of this plant [7]. However, in the methanolic extract as well as the aqueous extract of this plant we could detect more of those derivatives and this fact is of ethnopharmacological relevance since the herbal tea is usually the way this plant is consumed since pre-Hispanic times. For instance, the main compound in the aqueous extract was an opened filifolinoic acid, peak ([M − H]^−^ ion at *m*/*z* 307.1553, C_17_H_23_O_5_^−^). However, methyl ester derivatives of filifoninolic acid or filifolinol [6,22] were not detected in the aqueous and methanolic extract, but were previously detected in the resin exudate (chloroform extract) by us [7]. Thus peaks 9 ([M − H]^−^ ion at *m*/*z* 339.14542, C_17_H_23_O_7_^−^), peak 10 ([M − H]^−^ ion at *m*/*z* 323.15051, C_17_H_23_O_6_^−^), peak 34 ([M − H]^−^ ion at *m*/*z* 307.15491, C_17_H_23_O_5_^−^), peak 18 ([M − H]^−^ ion at *m*/*z* 321.13434, C_17_H_21_O_6_^−^), peak 19 ([M − H]^−^ ion at *m*/*z* 305.13947, C_17_H_21_O_5_^−^), peak 52 ([M − H]^−^ ion at *m*/*z* 289.14450, C_17_H_21_O_4_^−^), and peak 46 ([M − H]^−^ ion at *m*/*z*: 289.1449038, C_17_H_21_O_4_^−^), which were identified as opened 2,5-dihydroxy-filifolinoic acid, opened 5-hydroxy-filifolinoic acid, opened filifolinoic acid, 2,5-dihydroxy-filifolinoic acid, 5-hydroxy-filifolinoic acid, filifolinoic acid, and 4,5-dihydroxy-3-geranyl-benzoic acid, respectively [7].

**Figure 3:**
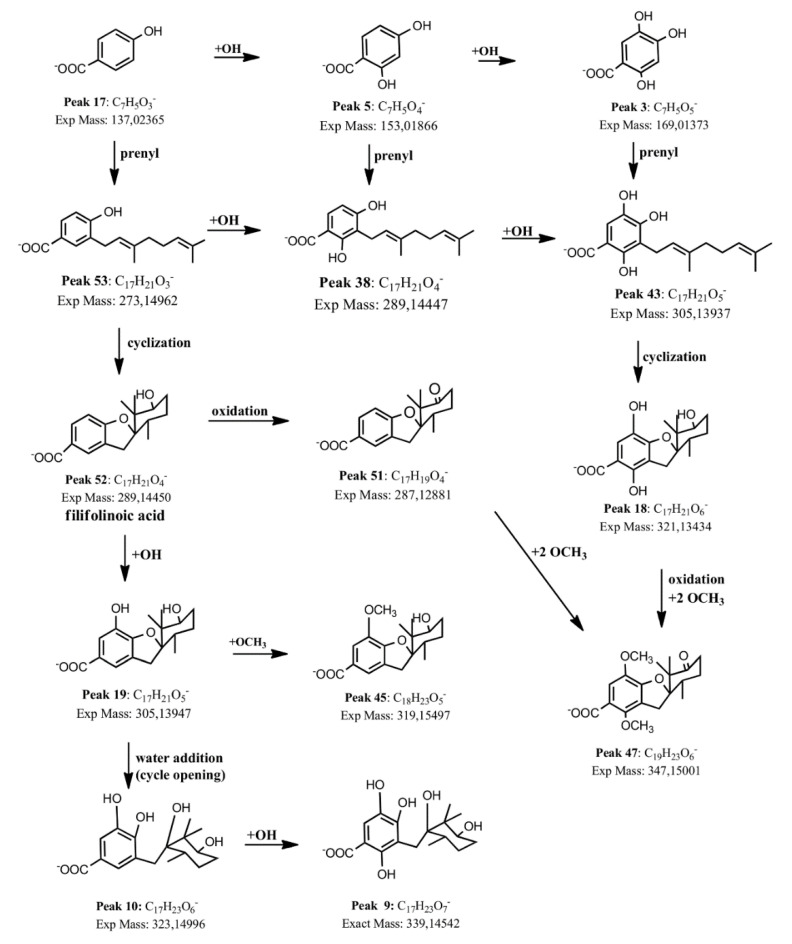
Proposed biosynthetic pathway for the benzoic acid derivatives detected in *H. taltalense.*

New detected benzoic acid derivatives were: peak 32, which was identified as 2,4,5-trihydroxy-3-geranyl-benzoic acid ([M − H]^−^ ion at *m*/*z* 305.13937, C_17_H_21_O_5_^−^), peak 45 ([M − H]^−^ ion at *m*/*z*: 319.15497), which was identified as 5-methoxy-oxy-filifolinoic acid (C_18_H_23_O_5_^−^), peak 36 ([M − H]^−^ ion at *m*/*z*: 321.13440), which was identified as 2,5-dihydroxy-filifolinoic acid (C_17_H_21_O_6_^−^), peak 14 ([M − H]^−^ ion at *m*/*z*: 323.14987), which was identified as an opened 2-hydroxy-filifolinoic acid derivative (C_17_H_23_O_6_^−^), formed by the opening of the tetrahydrofurane ring by addition of a molecule of water, peak 22 (305.13950), which was identified as 2-hydroxy-filifolinoic acid (C_17_H_23_O_6_^−^), peak 27 ([M − H]^−^ ion at *m*/*z*: 305.13947), which was identified as its isomer, 6-hydroxy-filifolinoic acid (C_17_H_21_O_5_^−^, OH groups at C5 and C6), peak 38 ([M − H]^−^ ion at *m*/*z*: 289.14447), which was identified as 2,4-dihydroxy-3-geranyl-benzoic acid (C_17_H_21_O_4_^−^), peak 43 ([M − H]^−^ ion at *m*/*z*: 305.13934), which was identified 2,4,6-trihydroxy-3-geranyl-benzoic acid, and peak 53 ([M − H]^−^ ion at *m*/*z* 273.14962), which was identified as 2-hydroxy-3-geranyl-benzoic acid (C_17_H_21_O_3_^−^). In the same manner, peak 30 ([M − H]^−^ ion at *m*/*z*: 305.13931) was identified as another isomer of 5-hydroxy-filifolinoic acid (C_17_H_21_O_5_^−^) where the opened hydroxyl-geranyl-benzoic acid molecule cycled with the other free OH group placed in the position 2 of the aromatic ring forming a rearranged derivative with a free OH group at position C-4. Peaks 42, 47, 50, and 51 were identified as the oxidation product derivatives 6′-oxo-5-hydroxyfilifolinoic acid ([M − H]^−^ ion at *m*/*z*: 303.12375, C_17_H_19_O_5_^−^), 2,5-dimethoxy-6′-oxo-filifolinoic acid, 2,6-dimethoxy-6′-oxo-filifolinoic acid, (both with [M − H]^−^ ions at *m*/*z* 347.14999, C_19_H_23_O_6_^−^), and 6′-oxo-filifolinoic acid ([M − H]^−^ ion at *m*/*z* 287.12881, C_17_H_19_O_4_^−^), respectively. Figure 3 shows a proposed biosynthetic pathway between benzoic acid derivatives and Figure S1a–j supplementary material shows examples of full MS Orbitrap spectra with related structures. The shikimate biosynthetic pathway led to simple benzoic acids, such as peaks 3, 5, and 17 in the plant. Carbon–carbon coupling reactions (from peaks 17 to peak 53, from peak 5 to peak 38, and from peak 3 to peak 43, Figure 3) were performed by *C*-alkylation of the activated ortho position of the phenol with the activated geranyl moiety (geranyl pyrophospate). Oxidation was performed by oxidases, those enzymes were characterized as cytochrome P-450-dependent proteins, requiring NADPH and O_2_ cofactors (such reactions can be depicted in Figure 3, from peak 52 to peak 51, from peak 18 to peak 47, and from peak 53 to 38 and in turn to 43, and from peak 10 to peak 9). Methylation is usually performed by S-adenosylmethionine (SAM, reactions from peak 51 to peak 47, and from peak 19 to peak 45, Figure 3) [23].

#### 2.1.5. Flavonoids

Peaks 20, 33, and 40 with pseudo-molecular ions at *m*/*z* 287.05606, 285.07684, and 255.06621 were tentatively identified as the flavanones eriodictyol (C_15_H_11_O_6_^−^), sakuranetin (C_15_H_9_O_6_^−^), and pinocembrin (C_16_H_13_O_6_^−^), while peak 31 was identified as the flavanone naringenin (C_15_H_11_O_5_^−^), peak 26 as 3-hydroxyhesperetin (C_16_H_13_O_7_^−^), and peak 28 as pinostrobin (C_15_H_11_O_4_^−^). Peaks 21 and 23 were identified as the flavonols quercetin (C_15_H_9_O_7_^−^), and isorhamnetin (C_16_H_11_O_7_^−^). Several *O*-methylated derivatives of flavonoids were identified as well. Peaks 44, 39, and 35 were identified as the i-methylated quercetin derivatives: isorhamnetin 7-methyl ether (C_17_H_13_O_7_^−^) [24], and isorhamnetin 4′-methyl ether (C_17_H_13_O_7_^−^). Peak 37 was identified as 7-methoxykaempferol (rhamnocitrin or kaempferol 7-methyl ether, C_16_H_11_O_6_^−^ [25]) and peak 29 as kaempferol 4′,7-dimethyl ether (C_17_H_13_O_6_^−^) and peak 35 as kaempferol 3′,7-dimethyl ether (C_17_H_13_O_6_^−^). Peak 48 was identified as apigenin 7-methyl ether (C_16_H_11_O_5_^−^) and peak 49 as luteolin 4′,7-dimethyl ether (C_17_H_13_O_6_^−^).

**Figure 4:**
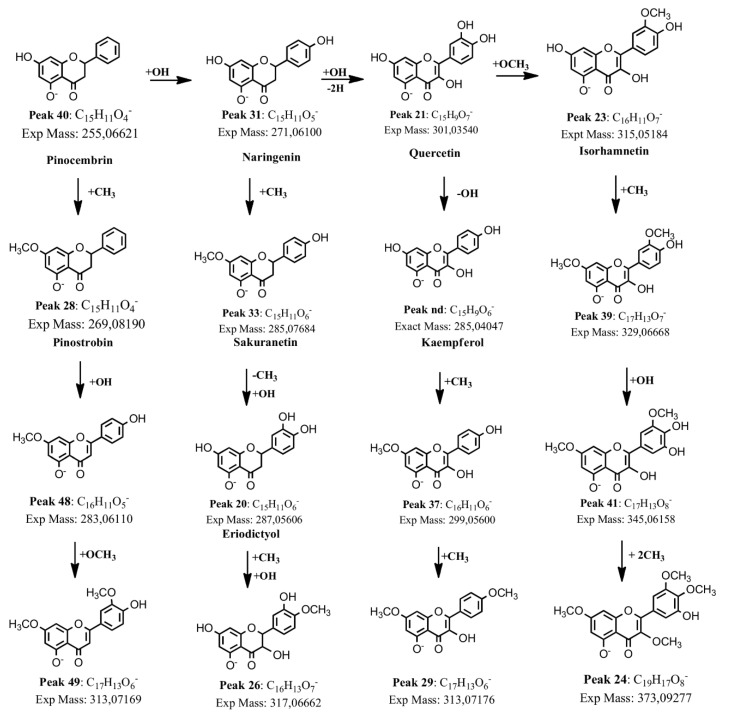
Proposed biosynthetic pathway between flavonoid derivatives detected in *H. taltalense.* nd: Compound not detected.

Finally, peak 24 was identified as myricetin 3′,4′,7-trimethyl ether (C_16_H_11_O_7_^−^) [24] and peak 41 as 3′,7-dihydroxymyricetin (C_17_H_13_O_8_^−^). Peak 12 with a pseudo-molecular ion at 593.15045 was identified as the only detected flavonol glycoside kaempferol 3-rutinoside (C_27_H_29_O_15_^−^) [26]. Figure 4 shows a biosynthetic pathway proposed for flavonoid derivatives.

### 2.2. Isolation and Identification of Major Compounds

From the methanolic extract of *H. taltalense* the following compounds were isolated: pinostrobin **1**, pinocembrin **2**, sakuranetin **3,** rhamnocitrin **4,** and 4,5 dihydroxy-3-geranyl-benzoic acid **5** (Figure 5) using a combination of chromatographic techniques (see experimental). Major isolated compounds were identified by UV and HR-MS spectra as well as 1D and 2D NMR spectra (Supplementary Material, Table S1 and Figures S2–S22). The five major compounds were present in both extracts, the aqueous and the methanolic extract.

Compound **1**, peak 28: 5-hydroxy-7-methoxy-flavanone, (pinostrobin). Colorless crystals, m.p. 119.7–120.0 °C. [M − H]^−^: 269.08190, (calcd for: C_15_H_11_O_4_^−^ = 269.08193). ^1^H NMR (300 MHz, CDCl_3_) δ ppm: 2.84 (1H, dd, *J* = 17.2 and 3.1 Hz), 3.11 (1H, dd, *J* = 13.0 and 17.2 Hz), 3.80 (s, OCH_3_), 5.44 (1H, dd, *J* = 3.1 and 13.0 Hz), 6.10 (1H, d, *J* = 2.3 Hz), 6.09 (1H, d, *J* = 2.3 Hz), 7.47 (5H, m), 12.11 (1H, s, OH). ^13^C NMR (100.15 MHz, CDCl_3_) δ ppm: 55.91 (OCH_3_), 79.14 (C-2), 42.75 (C-3), 196.16 (C-4), 162.44 (C-5), 94.62 (C-6), 167.94 (C-7), 94.13 (C-8), 163.87 (C-9), 102.77 (C-10), 137.86 (C-1), 126.96 (C-2), 129.16 (C-3), 126.96 (C-4), 129.16 (C-5), 126.96 (C-6). These data agree with the literature [27,28]. Furthermore, the X-ray crystal structure of this compound was already published by us [29].

Compound **2**, peak 40: 5,7′-dihydroxyflavanone (pinocembrin). Colorless crystals, m.p. 195–196 °C; (−) HRESIMS: [M−H]^−^ = 255.06621 (calcd for: C_15_H_11_O_4_^−^ = 255.06628). ^1^H NMR (Bruker advance, Germany, 400 MHz, CDCl_3_) δ ppm: 7.52 (2H, m, H-2′/H-6′), 7.41 (2H, m, H-3′/H-4′/H-5′), 5.95 (1H, d, u = 2.2 Hz, H-6), 5.91 (1H, d, *J* = 2.2 Hz, H-8), 5.47 (1H, dd, *J* = 12.7, 3.1 Hz, H-2), 3.10 (1H, dd, *J* = 17.1, 13.0 Hz, H-3a), 2.79 (1H, dd, *J* = 17.1, 3.0 Hz, H-3b). ^13^C NMR (100.15 MHz, CDCl_3_) δ: 196.8 (C=O), 164.9 (C-7), 163.8 (C-5), 163.6 (C-8a), 127.4 (C-4′), 130.9 (C-1′), 128.1 (C-2′/C-6′), 125.9 (C-3′/C-5′), 102.8 (C-4a), 95.9 (C-6), 94.6 (C-8), 78.9 (C-2), 42.6 (C-3).

Compound **3**, peak 33: 4′,5 -Dihydroxy-7-methoxyflavanone (sakuranetin). Colorless crystals, m.p. 152–153 °C; (−) HRESIMS: [M−H]^−^ = 285.07684 (calcd for: C_16_H_13_O_5_^−^ = 285.07685). ^1^H NMR (Bruker advance, Germany, 400 MHz, CDCl_3_) δ ppm: 7.26 (2H, d, *J* = 8.5 Hz, H-2′/H-6′), 6.83 (2H, d, *J* = 8.5 Hz, H-3′/H-5′), 6.01 (1H, d, *J* = 1.6 Hz, H-6), 6.02 (1H, d, *J* = 1.6 Hz, H-8), 5.32 (1H, dd, *J* = 13.0 and 3.0 Hz, H-2), 3.77 (3H, s, OCH_3_), 3.08 (1H, dd, *J* = 17.2 and 13.0 Hz, H-3a), 2.73 (1H, dd, *J* = 17.2 and 3.0 Hz, H-3b). ^13^C NMR (100.15 MHz, CDCl_3_) δ: 196.5 (C=O), 168.4 (C-4′), 164.5 (C-7), 163.0 (C-5), 156.5 (C-8a), 130.9 (C-1′), 128.5 (C-2′/C-6′), 116.2 (C-3′/C-5′), 103.5 (C-4a), 95.2 (C-6), 94.7 (C-8), 79.4 (C-2), 55.3 (OCH_3_), 43.2 (C-3). These data are in agreement with the literature [30]. Furthermore, the crystal structure of this compound was already published by us [31].

Compound **4**, peak 37: 3,5,4′trihydroxy-7-methoxy-flavone, (rhamnocitrin, kaempferol-7 methyl ether). Colorless crystals, m.p. 248–249 °C; (−) HRESIMS: [M − H]^–^ = 299.05600 (calcd for: C_16_H_11_O_6_^−^ = 299.05611). ^1^H NMR (Bruker advance, Germany, 400 MHz, CDCl_3_) δ ppm: 7.99 (2H, d, *J* = 13.8 Hz, H-5′/H-3′), 6.94 (2H, d, *J* = 12.0 Hz, H-2′/H-6′), 6.42 (1H, d, *J* = 2.1 Hz, H-8), 6.21 (1H, d, *J* = 2.1 Hz, H-6), 3.77 (3H, s, OCH_3_). ^13^C NMR (100.15 MHz, CDCl_3_) δ: 178.9 (C=O), 164.6 (C-7), 162.1 (C-5), 160.3 (C-2), 157.2 (C-8a), 157.0 (C-4′), 138.1 (C-3), 129.9 (C-3′/C-5′), 121.3 (C-1′), 115.3 (C-2′/C-6′), 104.9 (C-4a), 98.3 (C-6), 93.6 (C-8), 59.2 (OCH_3_). These data agree with the literature [27,28,32]

Compound **5**, peak 46: 4,5 dihydroxy-3-geranyl-benzoic acid. Colorless crystals, m.p. 131–132 °C; (−) HRESIMS: [M − H]^–^ = 289.1437 (calcd for: C_17_H_22_O_4_^–^ = 289.1445). ^1^H NMR (Bruker advance, Germany, 400 MHz, CDCl_3_) δ ppm: 7.40 (1H, d, *J* = 1.9 Hz, H-2), 7.35 (1H, d, *J* = 1.9 Hz, H-6), 5.34 (1H, td, *J* = 7.3, 1.1 Hz, H-2′), 5.10 (1H, ddd, *J* = 6.8, 3.9, 1.2 Hz, H-6^′^), 3.34 (2H, d, *J* = 6.7 Hz, H-1′), 2.07 (4H, m, H-4′ and H-5′), 1.72 (3H, s, Me-9′), 1.68 (3H, s, Me-8′), 1.58 (3H, s, Me-10′). ^13^C NMR (100.15 MHz, CDCl_3_) δ: 169.3 (-COOH), 148.1 (C-4), 144.0 (C-5), 135.8 (C-3’), 130.8 (C-7’), 127.7 (C-3), 124.0 (C-2’), 122.8 (C-2), 122.1 (Me-6′), 120.1 (C-1), 113.7 (C-6), 39.5 (C-4′), 27.5 (C-1′), 26.3 (C-5′), 24.5 (Me-8′), 16.4 (Me-10′), 14.9 (Me-9′). These NMR data is coincident with those previously reported by us [7].

### 2.3. Antioxidant Activity of H. taltalense

The results showed that the high polyphenols (TPC) and flavonoids content (TFC) in extracts agreed with its important antioxidant capacity (Table 1). The quantification of TPC and TFC in *H. taltalense* demonstrated that the methanolic extract contained the higher number of polyphenols and flavonoids than the aqueous extract. The extracts provided a dose-dependent antiradical activity inhibiting the radical DPPH and ABTS. The ferric reducing-antioxidant power (FRAP) assay results of *H. taltalense* showed that the methanolic and aqueous extract possessed high reducing power.

### 2.4. Role of the Endothelium in the Vascular Relaxation of H. taltalense

**Figure 6:**
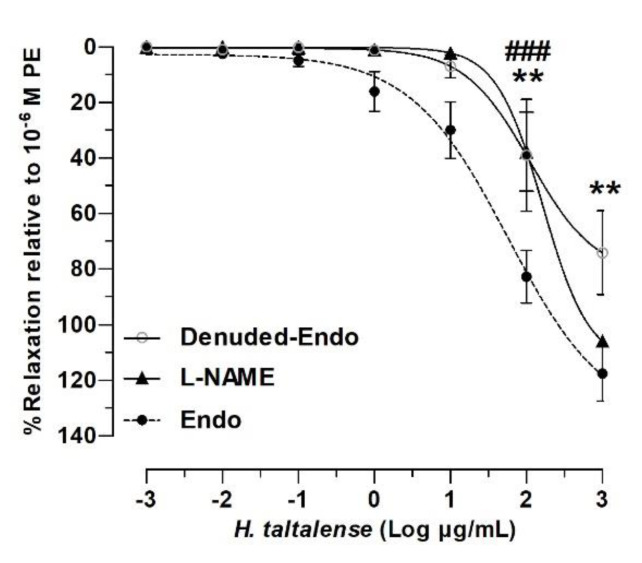
*H. taltalense* causes relaxation in rat aorta via the endothelium-dependent pathway. Aortic rings were pre-contracted with 10^−6^ M PE, and cumulative concentrations of aqueous extract (0.001–1000 mg/mL) was added in bath. Protocol was repeated in intact rat aorta (Endo), in denuded-endothelium aorta (Denuded-Endo) and in pre-incubated tissue with 10^−4^ M L-NAME. Data are the average standard error of the mean (SEM) of 3 independent experiments. ** *p* < 0.01 (Denuded-Endo), ^###^ *p* < 0.001 (L-NAME) vs. Endo.

The vascular relaxation observed suggests that *H. taltalense* would produce a potential hypotensive effect. In intact aorta the relaxation with 2 log mg/mL (100 μg/mL) aqueous extract was 83% ± 9%, while in denuded-endothelium aorta significantly decreased (39% ± 20%, *p* < 0.01; Figure 6).

Moreover, pre-incubation of the tissue with L-NAME inhibited the nitric oxide synthase (NOS), and the result showed that nitric oxide (NO) pathway is involved in the vascular response to *H. taltalense.* L-NAME significantly decreased the 2 log mg/mL (100 μg/mL) extract-induced relaxation compared to intact aorta (38% ± 14%; *p* < 0.001). The half-maximal inhibitor concentration (IC_50_) varied significantly in the absence of endothelium, and in the presence of L-NAME (164 ± 2 μg/mL Denuded-Endo and 112 ± 2 μg/mL L-NAME) compared to Endo (58 ± 2 μg/mL Endo; *p* < 0.001).

These findings suggest that vascular relaxation to *H. taltalense* is dependent on endothelium and the generation of NO. Interestingly, a low concentration (100 μg/mL) caused an endothelial effect dependent on nitric oxide, while a high concentration (1000 μg/mL) was independent. The 53 metabolites described in the extract of *H. taltalense* may explain this difference, but from the physiological and pharmacological point of view it is more important to note that a low concentration of the extract produces an endothelial nitric oxide-dependent effect.

### 2.5. H. taltalense Decreases the Contractile Response to KCl and PE

**Figure 7:**
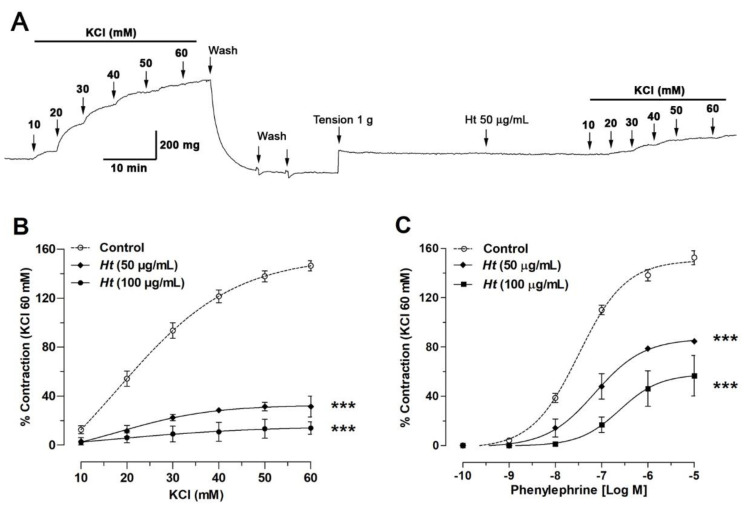
*H. taltalense* reduces the vascular contractile response in intact aorta. Original record of the vascular effect of *H. taltalense* in intact rat aorta (**A**). Tissue was contracted with accumulative concentrations of KCl (10–60 mM). Subsequently, a similar protocol was repeated in aortic rings pre-incubated with 50 μg/mL *H. taltalense* (Ht) for 20 min. Aortic rings were pre-incubated with different concentration of extract Ht (50 and 100 μg/mL) and accumulative concentrations of KCl (**B**) or phenylephrine (**C**) were added. Data are SEM of 4 independent experiments. *** *p* < 0.001 vs. control.

In the next experiment, we confirmed that relaxation induced by the aqueous extract was not an isolated vascular event and different from vascular contraction. Thus, the pre-incubation with *H. taltalense* significantly decreased the vascular contractile response to KCl: 146% ± 4% control vs. 31 ± 4% with 50 μg/mL Ht; *p* < 0.001 (Figure 7). Similar result was obtained with PE: 153% ± 6% control vs. 85% ± 1% with 50 μg/mL Ht; *p* < 0.001.

The half-maximal inhibitor concentration (EC_50_) did not vary significantly the vascular response to KCl in the absence (18 ± 9 mM control) or the presence of 50 μg/mL extract (23 ± 8 mM). However, the vascular response to PE in the absence (320 ± 87 μM control) or the presence of 50 μg/mL extract (719 ± 72 μM) was significantly different (*p* < 0.05).

Reduction of the contractile response to KCl would indicate that *H. taltalense* counteracts the KCl-induced depolarization in the plasma membrane. In response to depolarization of the membrane, extracellular Ca^2+^ enters the cell through voltage-gated calcium channels (VGCC) and vascular contraction occurs [33].

On the other hand, the reduction of contractile response to PE postulates that *H. taltalense* reduces the PE-induced pharmacological contraction. PE is a non-catecholamine alpha-1 agonist that produces dose-dependent vasoconstriction, involving the intracellular stores, in addition to influx of extracellular Ca^2+^. Increase of the vascular tone induced by PE is initiated by the opening IP3-sensitive sarcoplasmic reticulum channels, and maintained by repetitive Ca^2+^ waves from intracellular stores [34]. In addition, other calcium channels participate, such as VGCC [35].

### 2.6. Isolated Compounds from H. taltalense Causes Relaxation in Rat Aorta

**Figure 8:**
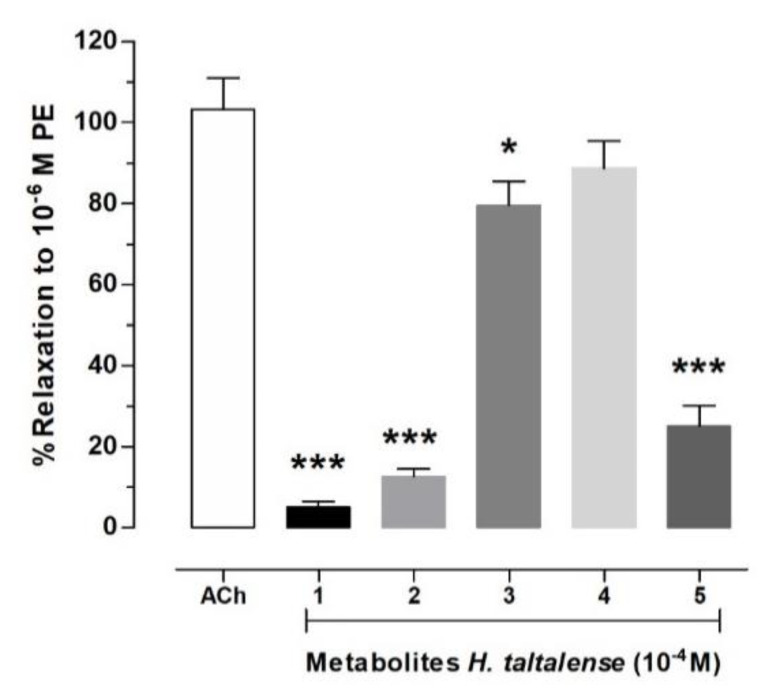
Isolated compounds from *H. taltalense* produced relaxation in intact rat aorta. Vascular relaxation of pinostrobin (**1**), pinocembrin (**2**), sakuranetin (**3**), rhamnocitrin (**4**), and 4,5 dihydroxy-3-geranyl-benzoic acid (**5**) were compared to ACh. Aortic rings were pre-contracted with 10^−6^ M PE, and then, the metabolites or ACh (10^−4^ M) was added in organ bath. Data are SEM of 3 independent experiments. * *p* < 0.05; *** *p* < 0.001 vs. ACh.

Five bioactive molecules were isolated from the methanolic extract of *H. taltalense* as described above. These compounds were present in both extracts, the aqueous and the methanolic extract. To evaluate the vascular relaxation of the isolated compounds: pinostrobin (**1**), pinocembrin (**2**), sakuranetin (**3**), rhamnocitrin (**4**), and 4,5 dihydroxy-3-geranyl-benzoic acid (**5**), we compared them to ACh (Figure 8; Purity: ± 95% by HPLC analysis). Only sakuranetin (**3**; 80% ± 6%) and rhamnocitrin (**4**; 89% ± 7%) possessed an important vascular relaxation in intact rat aorta, like ACh (103% ± 8%).

**Figure 9:**
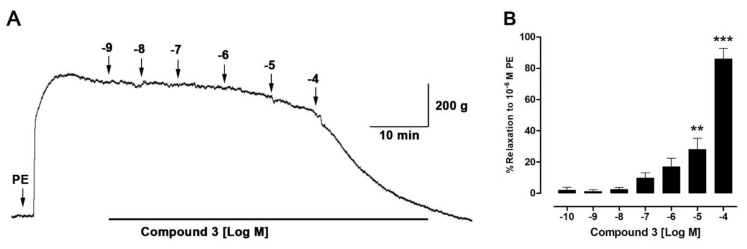
Sakuranetin causes vascular relaxation in intact aorta. Original record of the vascular effect of sakuranetin (compound **3**) in intact rat aorta (**A**). Tissue was pre-contracted with 10^−6^ M PE, and then, the cumulative concentrations of sakuranetin (10^−9^–10^−4^ M) were added in the bath (**B**). Data are the SEM of 3 independent experiments. ** *p* < 0.01 and *** *p* < 0.001 vs. 10^−9^ M compound **3**.

When chemical structures of the compound 1 (pinostrobin; 5-hydroxy-7-methoxy-flavanone) and 3 (sakuranetin; 4′,5 -dihydroxy-7-methoxyflavanone) were compared, we discovered that the increase of the vascular relaxation of compound **3** might be due to the inclusion of the free OH group at position 4′. In a previous study, we found that free OH groups in position 5 and 3′ were also important for the increase of the vascular relaxation in isolated metabolites from *N. ramosissima* [36]. On the other hand, the vascular relaxation effect between compounds **3** and **4** was not significantly different (Figure 8). Therefore, in the following experiment (Figure 9), we focused in figuring out the half maximal effective concentration (EC_50_) for compound **3** to produce vascular relaxation, which was 30.64 ± 6.5 μM.

As previously mentioned, the high content of polyphenols and flavonoids in the methanolic and aqueous extract of *H. taltalense* is associated with a great antioxidant capacity. It is postulated that flavanones are associated with a reduction risk of cardiovascular disease. This biological activity of flavanones would be mediated by vasodilator activity, improvement of vascular function, and antioxidant activity [37]. Hydroxyl groups in flavanones by scavenging free radicals are the cause of the antioxidant activity of the molecule [38]. Thus, antioxidant activity could readjust the unbalanced redox potential and pro-oxidant signaling systems in the cell, in such a way, balancing intracellular metabolism through regulation of receptors and ionic channels [39,40].

## 3. Materials and Methods

### 3.1. Chemicals and Plant Material

HPLC-MS Solvents including acetonitrile, methanol, hexane, and ethyl acetate were from Merck (Santiago, Chile). Sephadex LH-20 was obtained from Pharmacia Fine Chemicals (Piscataway, NJ, USA). Water was purified in a Millipore water purification system (Milli-Q, Merck Millipore, Santiago, Chile). Medium pressure chromatography: medium pressure lab pumps (QG 150, FMI, Syosset, NY, USA) coupled to medium pressure columns (Ace glass Inc., Vineland, NJ, USA). TLC: Silica gel 60 F_254_ precoated plates, silica gel 60 G (200 µm), silica gel 60 H (55 µm), and formic acid from Merck (Darmstadt, Germany). Mono (^1^H- and ^13^C-) and bidimensional (HSQC, HMBC, COSY, and Distortionless Enhancement by Polarization Transfer DEPT) NMR spectra: Bruker Avance 400 spectrometer (Bruker, Fallanden, Switzerland): δ in ppm relative to Me_4_Si as internal standard, *J* in Hz. The melting points were measured in a Stuart Scientific melting point SMP3 device (Bibby, London, UK). Standards for HPLC (purity up to 95%) were purchased from extrasynthese (Genay, France), or Sigma-Aldrich (Piscataway, NJ, USA). L-phenylephrine hydrochloride (PE), acetylcholine chloride (ACh), and N^G^-nitro-l-arginine methyl ester (L-NAME), which were bought from Sigma-Aldrich (St Luis, MO, USA). The aqueous extract was dissolved in physiological solution and metabolites isolated from methanolic extract were dissolved in DMSO (0.1% final concentration).

### 3.2. Plant Material 

*Heliotropium taltalense* (Phil.) I. M. Johnst was collected at Paposo Valley, II region, Northern Chile in April 2016 and identified by the botanist Alicia Marticorena (University of Concepción, Concepción, Chile). A voucher herbarium specimen was deposited at the Laboratorio de Productos Naturales, Universidad de Antofagasta, Antofagasta, Chile, with the number HT20160415.

### 3.3. Extraction and Isolation

Approximately 200 g of the dried plant was pulverized in a mortar and then extracted with 500 mL of HPLC-MS grade methanol in the dark (three times) in an ultrasonic bath for one hour each time. The solutions were combined and evaporated to dryness under reduced pressure (40 °C) to give 5.32 g of *H. taltalense* methanolic extract. For the preparation of the aqueous extract, 2 g of the pulverized plant was added distilled water (250 mL) at 45 °C and left to stand for 12 h. The plant material was then filtered, and the solution lyophilized to give 0.47 g of material for further analysis of the constituents. In addition, for the isolation of the main compounds, the methanolic extract (5 g) was adsorbed in silica gel 60 G (50 g) and slurred onto the top of a column containing silica gel 60 H (0.8 kg), and chromatographed using a medium pressure pump with an isocratic eluent (*n*-hexane–EtOAc 9:1, 5 mL/min), to obtain eight fractions (A to H). Fraction E was submitted to further purification through a silica-gel G open column eluting with mixtures of *n*-hexane/EtOAc (0–100%) to afford compound **5** (12 mg). Fraction F was chromatographed using a medium pressure pump (silica gel 60 H, 80 g, using *n*-hexane-EtOAc 9:1, flow: 5 mL/min) to afford compound **1** (8 mg, yield: 0.004%). Fraction G was re-chromatographed using silica gel 60 H (150 g, isocratic mode, *n*-hexane-EtOAc 9:1, flow: 5 mL/min) to afford compounds **3** and **4** (15 and 22 mg, yield: 0.0075% and 0.011%, respectively), while fraction G was purified using permeation thorough Sephadex LH-20 (100 g) with methanol-water 7:3 as eluent in two successive steps to afford compound **2** (21 mg, yield: 0.0105%).

### 3.4. UHPLC-PDA-MS Instrument

For UHPLC-PDA-MS analysis, 5 mg of the methanolic extract and lyophilized tea were individually dissolved in 2 mL of methanol, filtered (PTFE filter) and 10 μL were injected in the instrument. A Thermo Scientific Dionex Ultimate 3000 UHPLC with Chromeleon 7.2 Software (Thermo Fisher Scientific, Darmstadt, Germany) hyphenated with a Thermo high resolution Q-Exactive focus mass spectrometer were used for the analysis. The chromatographic system was coupled to the MS with a HESI II source. Nitrogen was obtained using a Genius NM32LA generator (purity >99.999%, Peak Scientific, Billerica, MA, USA) and was employed as collision and damping gas. Mass calibration was performed once a day, in positive and negative modes, to ensure a working accuracy lower than 5 ppm in mass. Ultramark 1621 (Alfa aesar, London, UK), caffeine, *N*-butylamine, buspirone hydrochloride, sodium dodecyl sulfate, and taurocholic acid sodium salt (Sigma-Aldrich, Saint Louis, MO, USA) were used as calibration standards for positive ions and negative ions to calibrate the spectrometer. Standards dissolved in a mixture of methanol-acetic acid-acetonitrile-water (Merck, Darmstadt, Germany) were infused using a Chemyx Fusion 100 syringe pump (Thermo Fisher Scientific, Bremen, Germany). XCalibur 2.3 and Trace Finder 3.2 software (Thermo Fisher Scientific, San José, CA, USA) were used for UHPLC control and data processing, respectively. Q Exactive 2.0 SP 2 software was used for instrument control.

### 3.5. LC Parameters

Liquid chromatography was performed using an UHPLC C-18 column (Acclaim, 150 mm × 4.6 mm ID, 2.5 μm, Thermo Fisher Scientific, Bremen, Germany) operated at 25 °C. The detection wavelengths were 254, 280, and 320 nm, and PDA was recorded from 200 to 800 nm for peak characterization. Mobile phases were 1% formic aqueous solution (A) and 1% formic acid in acetonitrile (B). The gradient used was (time, min, % (B): (0.00 min, 3); (5.00 min, 3); (10.00 min, 23); (15.00 min, 33); (22.00 min, 60); (30.00 min, 60); (38.00 min, 5); and 12 min before next injection. The flow rate was 1.00 mL min^−1^, and the volume injected was 20 μL. Extracts dissolved in methanol were kept at 10 °C in the machine.

### 3.6. MS Parameters

The HESI II parameters were: sheath gas flow rate 75 units; auxiliary gas flow rate 20; capillary temperature: 400 °C; aux gas heater: 500 °C; spray voltage 2500 V (for ESI^−^); and S lens RF level 30. Full scan data (in negative mode) was acquired at a resolving power of 70,000 FWHM (full width half maximum) at *m*/*z* 200. A scan range of *m*/*z* 100–1000 was selected; the automatic gain control (AGC) was set at 3 × 10^6^ and the injection time was set to 200 ms. Scan-rate was 2 scans s^−1^. MS^n^ targeted analysis for confirmation purposes was performed using inclusion list of masses and retention times of the analytes, with a time window of 30 sec and with the Orbital trap spectrometer operating in the negative mode at 17,500 FWHM (*m*/*z* 200). The AGC target was set to 2 × 10^5^, with the maximum injection time of 20 ms. The precursor ions were filtered by the quadrupole, which operates at an isolation window of *m*/*z* 2. The fore vacuum, high vacuum, and ultrahigh vacuum were maintained at 2, 10^5^, and 10^10^ mbar, respectively. Higher-Energy Collisional Dissociation (HCD) cell collision energy was operated at 30 kv. Detection based on calculated exact mass and on retention time of target compounds. Full exact mass and retention time of target compounds presented in Table S1 (Supplemental Material). Mass tolerance window for the negative mode was set to 5 ppm.

### 3.7. Determination of Antioxidant Activity

In vitro antioxidant activity was determined using the methods described in Supplementary Materials. Several assays, such as total phenolic content (TPC), total flavonoids content (TFC), DPPH, FRAP, and ABTS were used to evaluate the methanolic and aqueous extract of *H. taltalense*.

### 3.8. Animals

The study used male Sprague Dawley rats (6–8 weeks old; *n* = 10) weighing between 170 and 200 g. The investigation was conducted in accordance with the local animal research committee of Universidad de Antofagasta (which approved the experimental procedure used in the present study, CEIC #135/2018). The animals were housed in plastic cages at a room temperature of 22–25 °C and humidity of 45–51% and had full access to tap water and food ad libitum. They were randomized and assigned into groups.

### 3.9. Isolation of Rat Aorta and Vascular Reactivity Essays

This procedure was performed based on the method previously described [41]. Animals were euthanized for cervical dislocation. The aortic rings were placed in an organ bath with Krebs-Ringer bicarbonate solution (KRB; 4.2 KCl, 1.19 KH_2_PO_4_, 120 NaCl, 25 NaHCO_3_, 1.2 MgSO_4_, 1.3 CaCl_2_, and 5 d-glucose, pH 7.4, 37 °C, 95% O_2_, and 5% CO_2_). To evaluate the effect of *H. taltalense* extracts on the contractile response to phenylephrine (PE, 10^−10^–10^−5^ M) or KCl (10–60 mM) the tissue was pre-incubated for 20 min prior to contraction. In another applied protocol, the relaxation capacity of the extract or isolated metabolites was determined. In this case, the tissue was pre-contracted with 10^−6^ M PE, and increasing concentrations of *H. taltalense* extracts or metabolites were added to the organ bath on the vascular plateau response. Previously in all experiments, the integrity of the vascular endothelium was evaluated with 10^−6^ M acetylcholine (ACh).

### 3.10. Statistical Analysis

The results obtained from these experiments were expressed as mean ± standard error of mean. Statistical analysis of the data was performed using analysis of variance (two-way ANOVA) where applicable followed by post-hoc Bonferroni test. In addition, the determination of the sensitivity (EC_50_ or IC_50_) was performed using nonlinear regression (sigmoidal) via Graph Pad Prism software, version 5.0. (GraphPad Software, Inc., La Jolla, CA, USA). Statistical significance is set at *p* = 0.05.

## 4. Conclusions

The extract for the endemic species *H. taltalense* showed significant vascular relaxation. This effect could be attributed to the presence of 53 compounds, which were detected by UHPLC-MS. Among the compounds detected, four were phenolic acids (peaks 8, 13, 15, and 25), thirteen were flavonoids (peaks 12, 20, 21, 23, 24, 26, 28, 29, 31, 33, 35, 37, and 40), four were organic saturated acids (peaks 1, 2, 6, 7, and 11), and twenty-five were benzoic acid derivatives (peaks 3–7, 9–10, 14,16–19, 22, 30, 32, 36, 38, 42, 43, 45, 46, and 50–53). Furthermore, five major compounds including four flavonoids and one geranyl benzoic acid derivative were isolated by column chromatography and showed vascular relaxation. Since extracts of *H. taltalense* produced vascular relaxation through an endothelium-dependent mechanism in rat aorta, and the compounds rhamnocitrin and sakuranetin also caused vascular relaxation similar to the extracts of *H. taltalense*, these pure compounds to some extent are likely responsible for vascular relaxation. Interestingly, both rhamnocitrin and sakuranetin were the most abundant (by its relative intensity of peaks) in the aqueous extract, which supports our hypothesis. However, the synergistic effect of all flavonoids could be also responsible for the vascular response of the extracts, and more research is needed to support the antihypertensive effects of the phenolic compounds present in this plant.

## Figures and Tables

**Figure 5:**
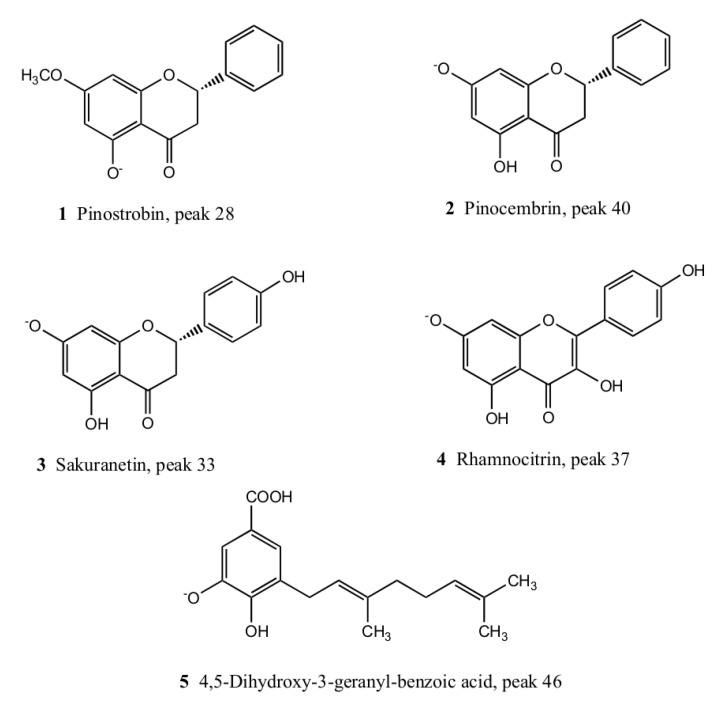
Isolated compounds from *H.* *taltalense* methanolic extract. 5-hydroxy-7-methoxy-flavanone (pinostrobin; **1**), 5,7′-dihydroxy-flavanone (pinocembrin; **2**), 4′,5 -dihydroxy-7-methoxy-flavanone (sakuranetin; **3**), 3,5,4′trihydroxy-7-methoxy-flavone, (rhamnocitrin; **4**), and 4,5 dihydroxy-3-geranyl-benzoic acid (**5**).

**Table 1:** Total phenolic content (TPC), total flavonoids content (TFC), DPPH, ABTS, and FRAP (ferric reducing-antioxidant power) were used to evaluate the methanolic (Ht MeOH) and aqueous extract (Ht Aq) of *H. taltalense*.

Extract	TPC	TFC	IC_50_ DPPH	IC_50_ ABTS	FRAP
Ht MeOH	1283 ± 175	743 ± 51	400 ± 2	66 ± 1	4067 ± 262
Ht Aq	75 ± 2	261 ± 3	595 ± 3	40 ± 0	2503 ± 435

The results are expressed as TPC in mg gallic acid equivalent/g dry extract, TFC in mg quercetin equivalent/g dry extract, IC_50_ DPPH and ABTS in μg/mL, and FRAP in mg trolox equivalent/g dry extract. Data are the average standard error of the mean (SEM) of 3 independent experiments.

## Supplementary Materials

The following are available online.
