# Supplementary material for: Chemical Fingerprinting, Isolation and Characterization of Polyphenol Compounds from Heliotropium taltalense (Phil.) I.M. Johnst and Its Endothelium-Dependent Vascular Relaxation Effect in Rat Aorta
Source: Molecules. 2020 Jul 8;25(14):3105. doi: 10.3390/molecules25143105 (PMC7397318; doi:10.3390/molecules25143105)
Supplement: Supplementary file 1 [file molecules-25-03105-s001.pdf]

Supplementary material

Table. S1. UHPLC PDA-HR-MS^n identification of metabolites in *H. taltalense* (methanolic and aqueous extract)

**Peak #**	**Retention time (min.)**	**UV max (nm)**	**Tentative identification**	**Formula [M-H]$^-$**	**Theoretical mass (*m/z*)**	**Measured mass (*m/z*)**	**Accuracy (*δ ppm*)**	**MS^n ions (*m/z*)**	**Extract**
1	1.52	-	malic acid*	$C_4H_5O_5^-$	133.01425	133.01352	-5.48		Me, Aq
2	1.63	-	citric acid*	$C_6H_7O_7^-$	191.01973	191.01926	-2.46		Me, Aq
3	1.83	280-330	2,4,5-trihydroxybenzoic acid	$C_6H_5O_5^-$	169.01425	169.01373	-3.07	125.02364 ($C_6H_5O_3^-$,M^- -CO_2)	Me, Aq
4	2.22	280-325	4-hydroxy-3,5-dimethoxybenzoic acid	$C_9H_9O_5^-$	197.04555	197.04515	-2.03	109.02856 ($C_6H_5O_2^-$)	Me, Aq
5	3.15	292-320	2,4-dihydroxybenzoic acid	$C_7H_5O_4^-$	153.01933	153.01866	-4.37	109.02855 ($C_6H_5O_2^-$, M^- - CO_2)	Me
6	3.12	-	dihydroxyheptanoic acid	$C_7H_{13}O_4^-$	161.08193	161.08122	-4.41	109.02856 ($C_6H_5O_2^-$)	Me
7	3.89	-	dihydroxyheptanoic acid glucoside	$C_{13}H_{23}O_9^-$	323.13476	323.13461	-0.46	135.04433 ($C_8H_7O_2^-$, M^- - CO_2)	Me
8	4.83	233-291	Caffeic acid*	$C_9H_7O_3^-$	179.03498	179.03448	-2.79	135.04433 ($C_8H_7O_2^-$, M^- - CO_2)	Me, Aq
9	8.23	235sh-289	Opened 2,5-dihydroxy-filifolinoic acid	$C_{17}H_{23}O_7^-$	339.14493	339.14542	1.44		Me, Aq

10	8.91	235sh-289	Opened 5-hydroxy-filifolinoic acid	$C_{17}H_{23}O_6^-$	323.15001	323.14996	-0.15		Me
11	9.2	210	tetrahydroxy-tetradecadienoic acid-O-glucoside	$C_{20}H_{33}O_{11}^-$	449.20284	449.20282	-0.04	138.04434 $C_8H_7O_2^-$	Me
12	9.51	266-354	Kaempferol- 3-O-rutinoside	$C_{27}H_{29}O_{15}^-$	593.15119	593.15045	-1.24		Me, Aq
13	9.55	327	Rosmarinic acid*	$C_{18}H_{15}O_8^-$	359.07724	359.07703	-0.58	161.02396 ($C_9H_5O_3^-$); 179.03428 ($C_9H_7O_4^-$) cafeic acid	Me, Aq
14	9.63	235sh-289	Opened 2-hydroxy-filifolinoic acid	$C_{17}H_{23}O_6^-$	323.15001	323.14987	-0.43		Me
15	9.74	292	ferulic acid	$C_{10}H_9O_4^-$	193.05063	193.05013	-2.59	133.02864 ($C_8H_5O_2^-$)	Me
16	11.11	235sh-289	opened filifolinoic acid	$C_{17}H_{23}O_5^-$	307.15510	307.15490	-3.29	161.02370 ($C_9H_5O_3^-$)	Me, Aq
17	11.55	280-310	p-hydroxybenzoic acid	$C_7H_5O_3^-$	137.02442	137.02365	-5.62		Me, Aq
18	12.01	235sh-289	2,5-dihydroxy-filifolinoic acid	$C_{17}H_{21}O_6^-$	321.13436	321.13434	-0.06	135.04430 ($C_8H_7O_2^-$)	Me, Aq
19	12.08	235sh-289	5-hydroxy-filifolinoic acid	$C_{17}H_{21}O_5^-$	305.13945	305.13947	0.07	135.04439 ($C_8H_7O_2^-$)	Me, Aq
20	12.32	282	Eriodictyol*	$C_{15}H_{11}O_6^-$	287.05611	287.05606	-0.17	135.04436 ($C_8H_7O_2^-$)	Me, Aq
21	12.45	254-354	Quercetin*	$C_{15}H_9O_7^-$	301.03538	301.03540	0.07	151.00285 ($C_7H_3O_4^-$)	Me, Aq
22	12.73	235sh-289	2-hydroxy-filifolinoic acid	$C_{17}H_{21}O_5^-$	305.13945	305.13950	0.16	161.02365 ($C_9H_5O_3^-$)	Me, Aq
23	13.52	254-354	Isorhamnetin*	$C_{16}H_{11}O_7^-$	315.05103	315.05184	2.57	271.02454 ($C_{14}H_7O_6^-$)	Me, Aq

24	13.75	255-355	Myricetin 3',4',7-trimethyl ether	$C_{16}H_{11}O_7^-$	373.09289	373.09277	-0.32	271.02454 ($C_{14}H_7O_6^-$)	Me, Aq
25	14.06	293	p-methoxyferulic acid	$C_{11}H_{11}O_4^-$	207.06628	207.06580	-2.31		Me, Aq
26	14.09	287	3-hydroxyhesperetin	$C_{16}H_{13}O_7^-$	317.06668	317.06662	-1.19		Me
27	14.17	235sh-289	6-hydroxy-filifolinoic acid	$C_{17}H_{21}O_5^-$	305.13945	305.13947	0.07	161.02353 ($C_9H_5O_3^-$)	Me, Aq
28	14.32	287	Pinostrobin*	$C_{15}H_{11}O_4^-$	269.08193	269.08190	-0.3		Me, Aq
29	14.65	266-362	Kaempferol 4',7-dimethyl ether	$C_{17}H_{13}O_6^-$	313.07176	313.07175	-0.03	161.02367 ($C_9H_5O_3^-$)	Me, Aq
30	15.61	235sh-289	4-hydroxy-filifolinoic acid	$C_{17}H_{21}O_5^-$	305.13945	305.13931	-0.46	161.02388 ($C_9H_5O_3^-$)	Me, Aq
31	16.25	224-287	Naringenin*	$C_{15}H_{11}O_5^-$	271.06120	271.06110	-0.54	119.04929 ($C_8H_7O^-$)	Me, Aq
32	17.23	266-300sh	2,4,5-trihydroxy-3-geranyl-benzoic acid	$C_{17}H_{21}O_5^-$	305.13945	305.13937	-0.26	122.03641 ($C_7H_6O_2^-$)	Me, Aq
33	18.02	285	Sakuranetin*	$C_{16}H_{13}O_5^-$	285.07685	285.07684	0.04		Me, Aq
34	18.32	222-268	opened filifolinoic acid	$C_{17}H_{23}O_5^-$	307.15510	307.15491	-0.62	161.02370 ($C_9H_5O_3^-$)	Me, Aq
35	18.50	223-329	Kaempferol 3',7-dimethyl ether	$C_{17}H_{13}O_6^-$	313.07176	313.07172	-0.45	161.02367 ($C_9H_5O_3^-$)	Me, Aq
36	18.67	223-321	2,5-dihydroxy-filifolinoic acid	$C_{17}H_{21}O_6^-$	321.13436	321.13440	0.12	161.02374 ($C_9H_5O_3^-$)	Me, Aq
37	19.42	266-354	Rhamnocitrin*	$C_{16}H_{11}O_6^-$	299.05611	299.05600	-0.37	255.02960 ($C_{14}H_7O_5^-$)	Me, Aq
38	19.78	266-300sh	2,4-dihydroxy-3-geranyl-benzoic acid	$C_{17}H_{21}O_4^-$	289.14453	289.14447	-0.21	119.04422 ($C_8H_7O^-$)	Me

39	19.48	255-355	Quercetin 3', 4-dimethyl ether	$C_{17}H_{13}O_7^-$	329.06665	329.06668	0.09	161.02367 ($C_9H_5O_3^-$)	Me, Aq
40	19.91	281	Pinocembrin*	$C_{15}H_{11}O_4^-$	255.06628	255.06621	-0.47	135.04431 ($C_8H_7O_2^-$)	Me, Aq
41	19.97	255-355	3',7-dihydroxymyricetin	$C_{17}H_{13}O_8^-$	345.06159	345.06158	-0.03		Me, Aq
42	20.00	235sh-289	6'-oxo-5-hydroxyfilifolinoic acid	$C_{17}H_{19}O_5^-$	303.12424	303.12375	-1.62	119.04964 ($C_8H_7O^-$)	Me, Aq
43	20.31	280-320sh	2,4,5-trihydroxy-3-geranyl-benzoic acid	$C_{17}H_{23}O_5^-$	305.13945	305.13934	-0.36	123.04421 ($C_7H_7O_2^-$)	Me, Aq
44	20.72	255-355	Quercetin 3,7-dimethyl ether or 7-methoxy-isorhametin	$C_{17}H_{13}O_7^-$	329.06665	329.06653	-0.36	299.01929 ($C_{15}H_7O_7^-$)	Me, Aq
45	21.00	235sh-289	5-methoxyoxy-filifolinoic acid	$C_{18}H_{23}O_5^-$	319.15510	319.15497	-0.41	241.12289 ($C_{16}H1_7O_2^-$)	Me, Aq
46	21.52	266-320sh	4,5-dihydroxy-3-geranyl-benzoic acid	$C_{17}H_{21}O_4^-$	289.14453	289.14444	-0.32	119.04422 ($C_8H_7O^-$)	Me, Aq
47	21.65	235sh-289	2,5-dimethoxy- 6'-oxo-filifolinoic acid	$C_{19}H_{23}O_6^-$	347.15001	347.14999	-0.06	177.01895 ($C_9H_5O_4^-$)	Me, Aq
48	22.12	268-330	Apigenin 7-methyl ether	$C_{16}H_{11}O_5^-$	283.06120	283.06110	-0.35	135.04440 ($C_8H_7O_2^-$)	Me, Aq
49	22.35	254-267	3',7-dimethoxyluteonin	$C_{17}H_{13}O_6^-$	313.07176	313.07169	-0.22	283.02451 ($C_{15}H_7O_6^-$)	Me, Aq
50	23.54	235sh-289	2,6-dimethoxy- 6'-oxo-filifolinoic acid	$C_{19}H_{23}O_6^-$	347.15001	347.14999	-0.06	177.01892 ($C_9H_5O_4^-$)	Me
51	24.12	235sh-289	6'-oxo-filifolinoic acid	$C_{17}H_{19}O_4^-$	287.12888	287.12885	-0.14	122.03628 ($C_7H_6O_2^-$)	Me
52	25.32	235sh-289	Filifolinoic acid	$C_{17}H_{21}O_4^-$	289.14453	289.14450	-0.10		Me

53	26.13	266-300sh	2-hydroxy-3-geranyl-benzoic acid	$C_{17}H_{21}O_3^-$	273.14957	273.14962	0.18		Me

*Identified by spiking experiments with authentic standards. Me: methanol, Aq: aqueous.

Fig S1a-j. Full MS spectra and structures of peaks 12 (a), 30 (b), 31 (c), 34 (d), 35 (e), 38 (f), 41 (g), 44 (h), 46 (i) and 51 (j).

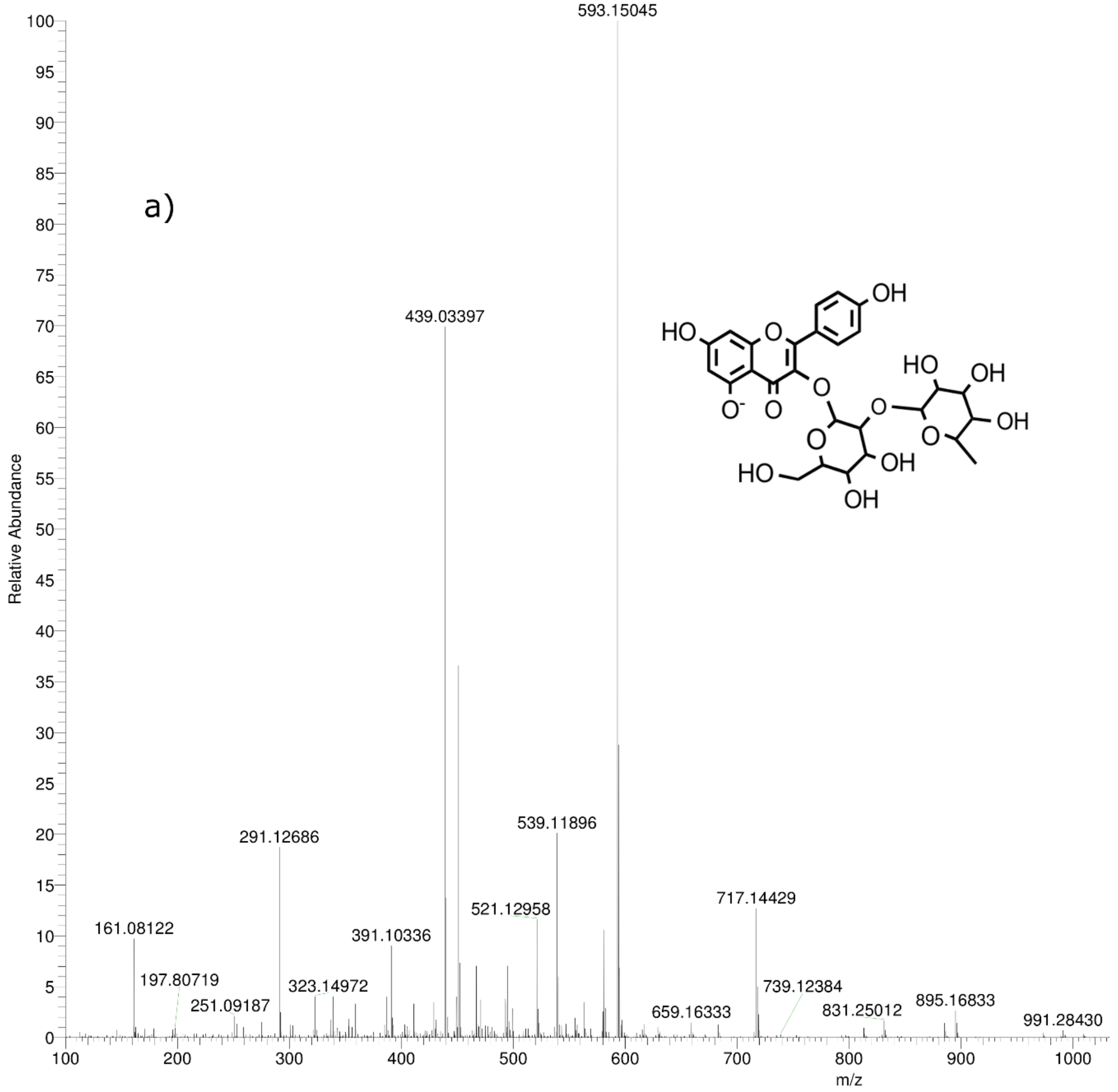

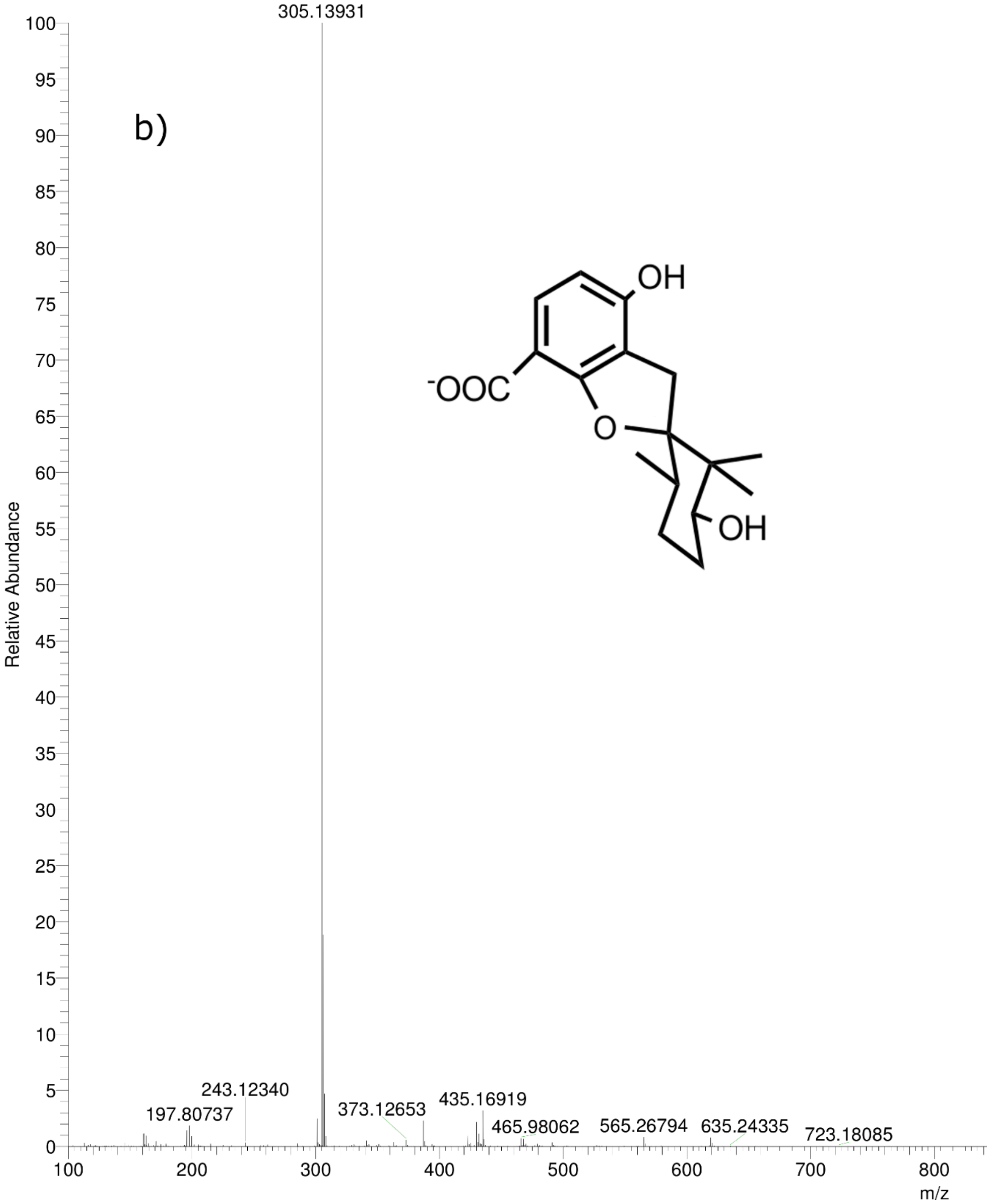

b)
305.13931
243.12340
197.80737
373.12653
435.16919
465.98062
565.26794
635.24335
723.18085
Relative Abundance
m/z
OH
-OOC
O
OH

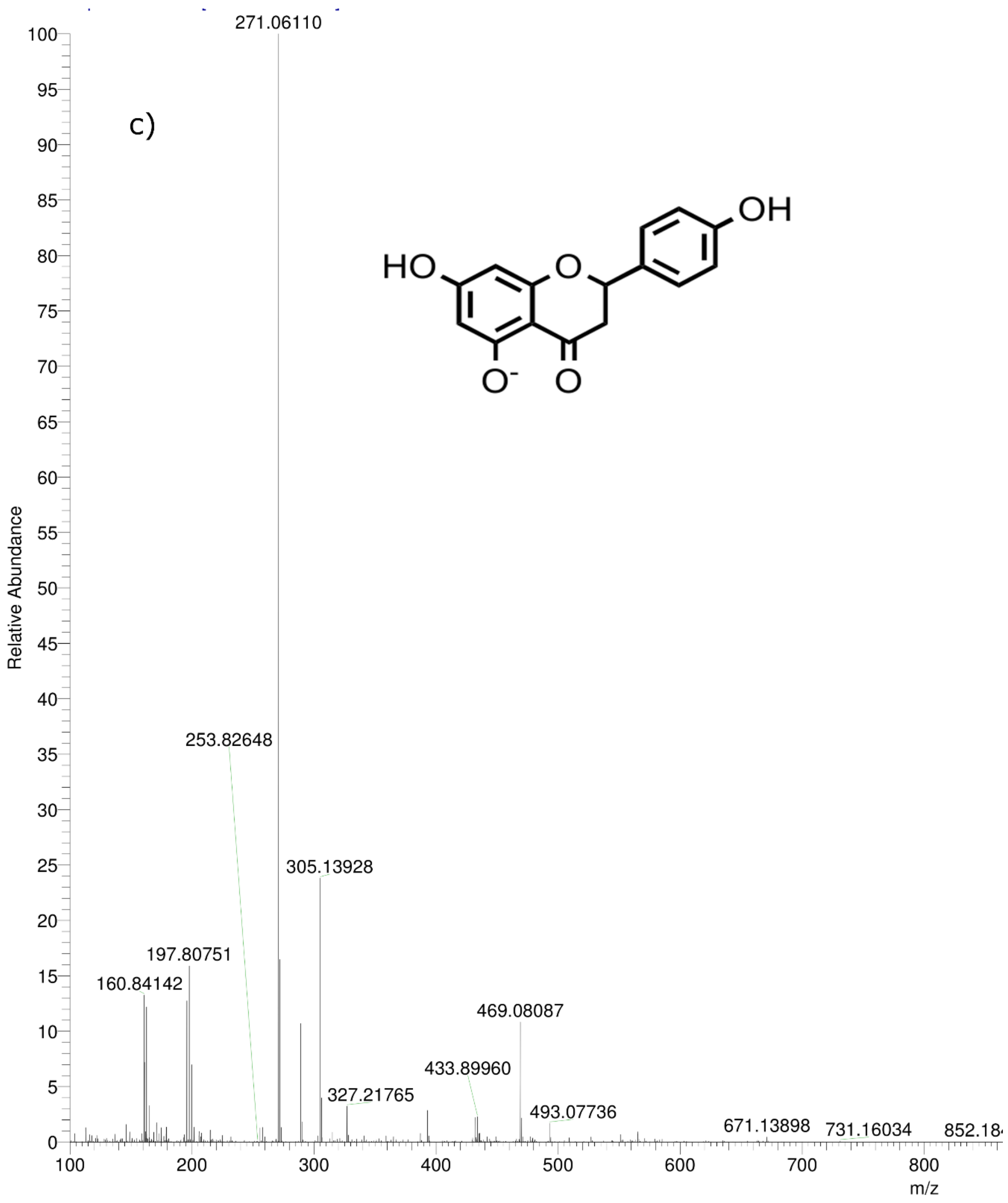

c)
271.06110
253.82648
305.13928
197.80751
160.84142
469.08087
433.89960
327.21765
493.07736
671.13898
731.16034
852.18
HO
O
OH
O⁻
O
Relative Abundance
m/z

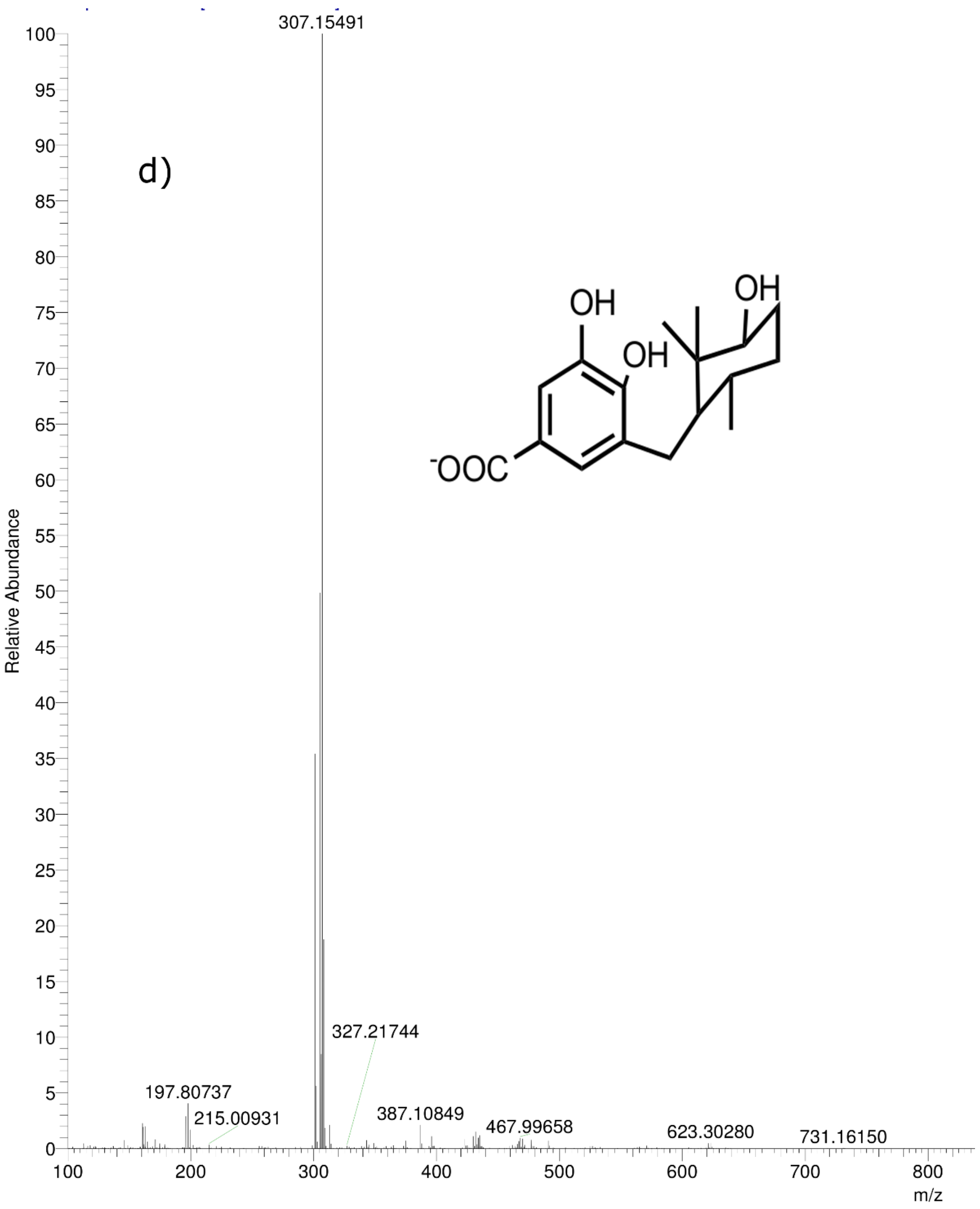

d)
307.15491
327.21744
197.80737
215.00931
387.10849
467.99658
623.30280
731.16150
Relative Abundance
m/z
OH
OH
OH
-OOC

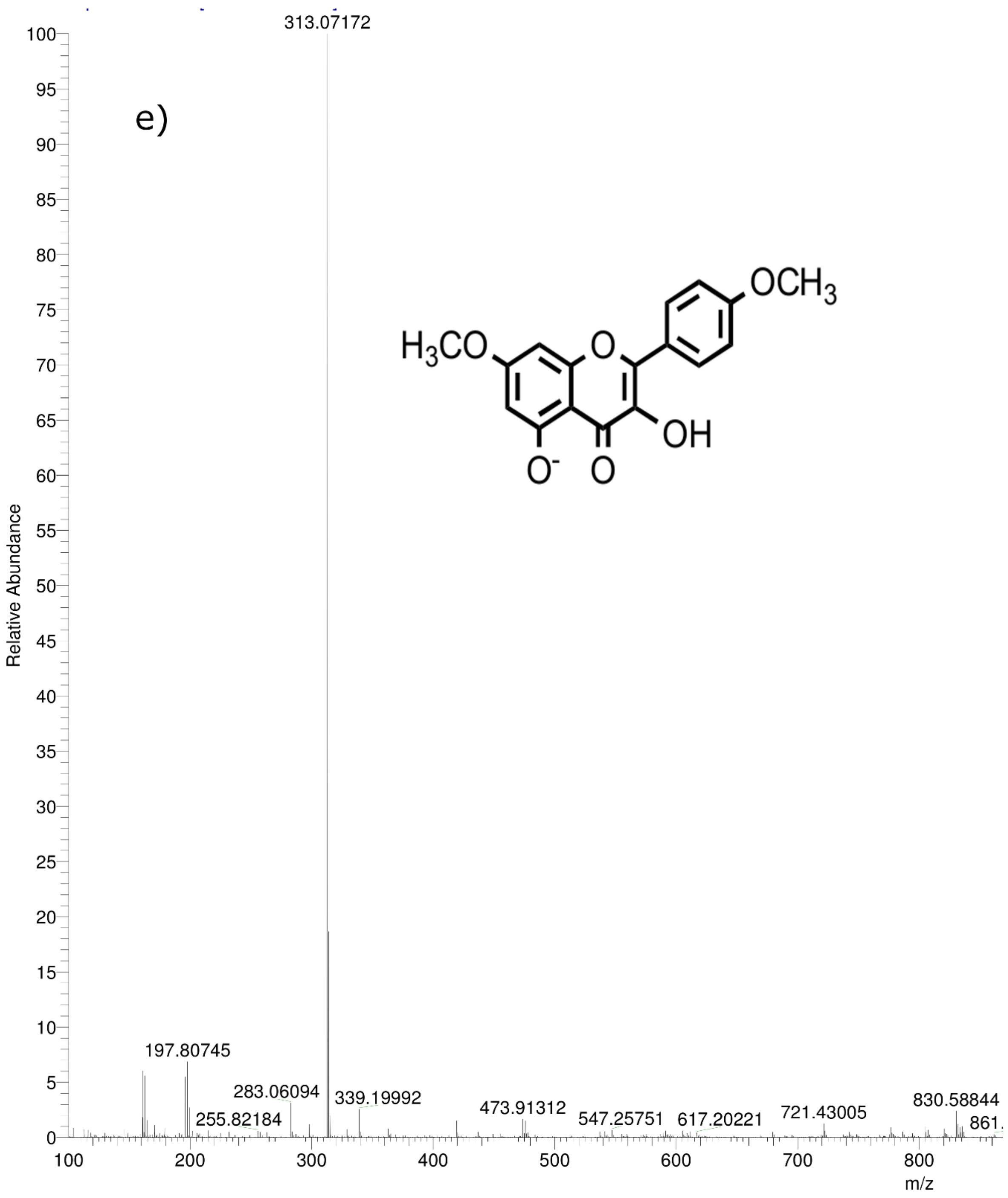

e)
313.07172
197.80745
283.06094
255.82184
339.19992
473.91312
547.25751
617.20221
721.43005
830.58844
861.
Relative Abundance
m/z
H3CO
OCH3
OH
O
O-

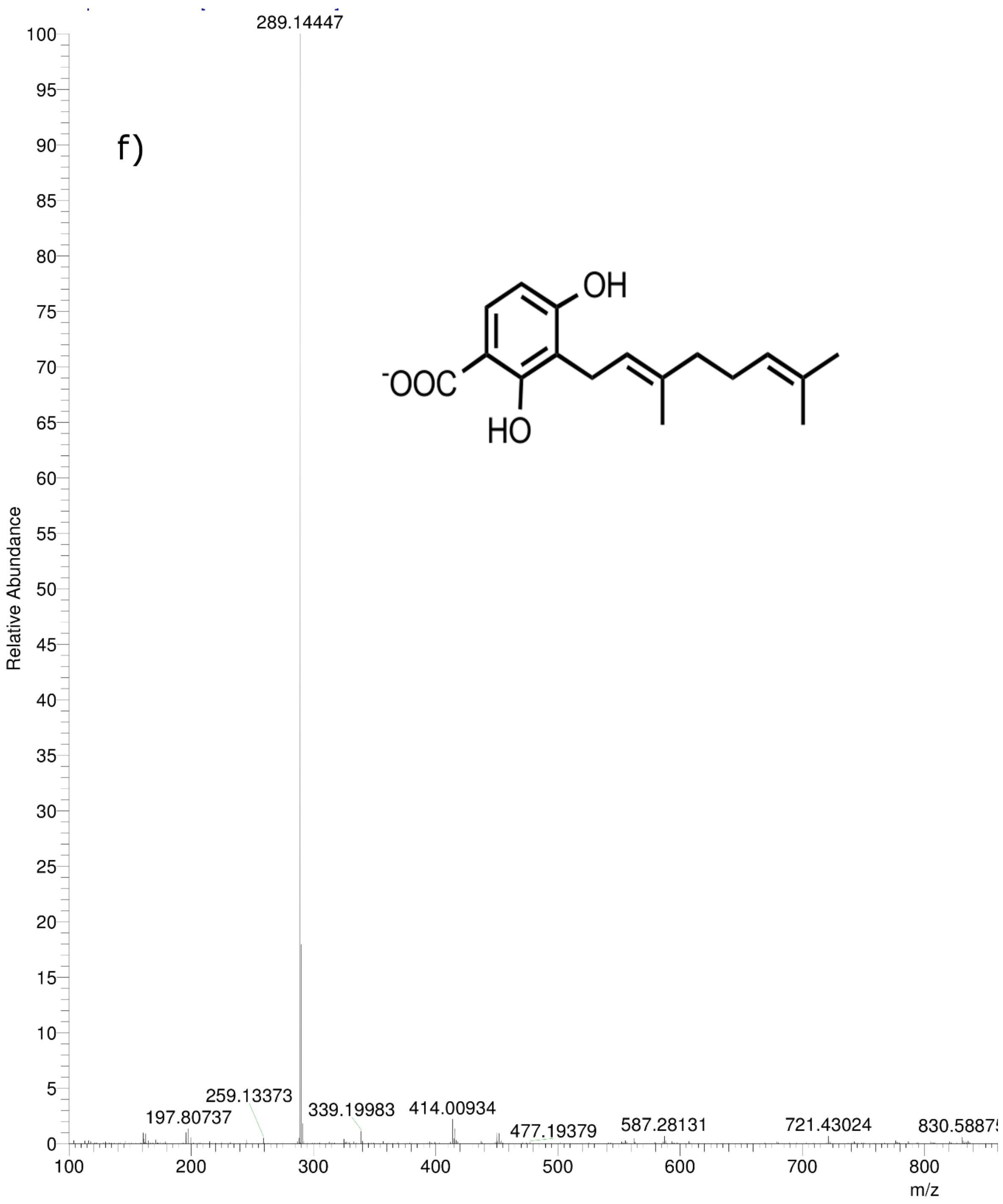

289.14447
f)
Relative Abundance
259.13373
197.80737
339.19983
414.00934
477.19379
587.28131
721.43024
830.5887
m/z
OH
⁻OOC
HO

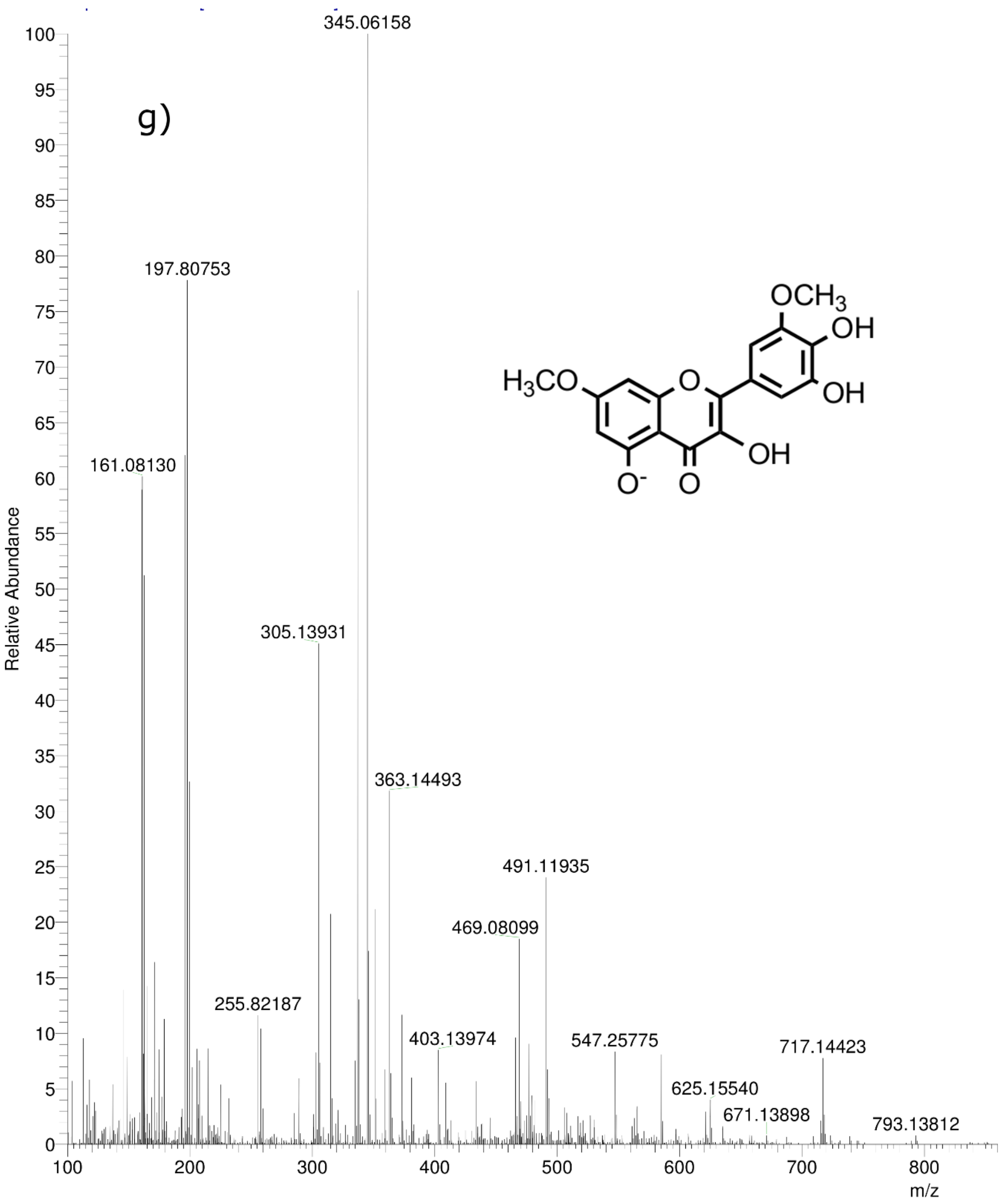
g)
345.06158
197.80753
161.08130
305.13931
363.14493
491.11935
469.08099
255.82187
403.13974
547.25775
717.14423
625.15540
671.13898
793.13812
Relative Abundance
m/z
OCH3
OH
H3CO
O
OH
OH
O-
O

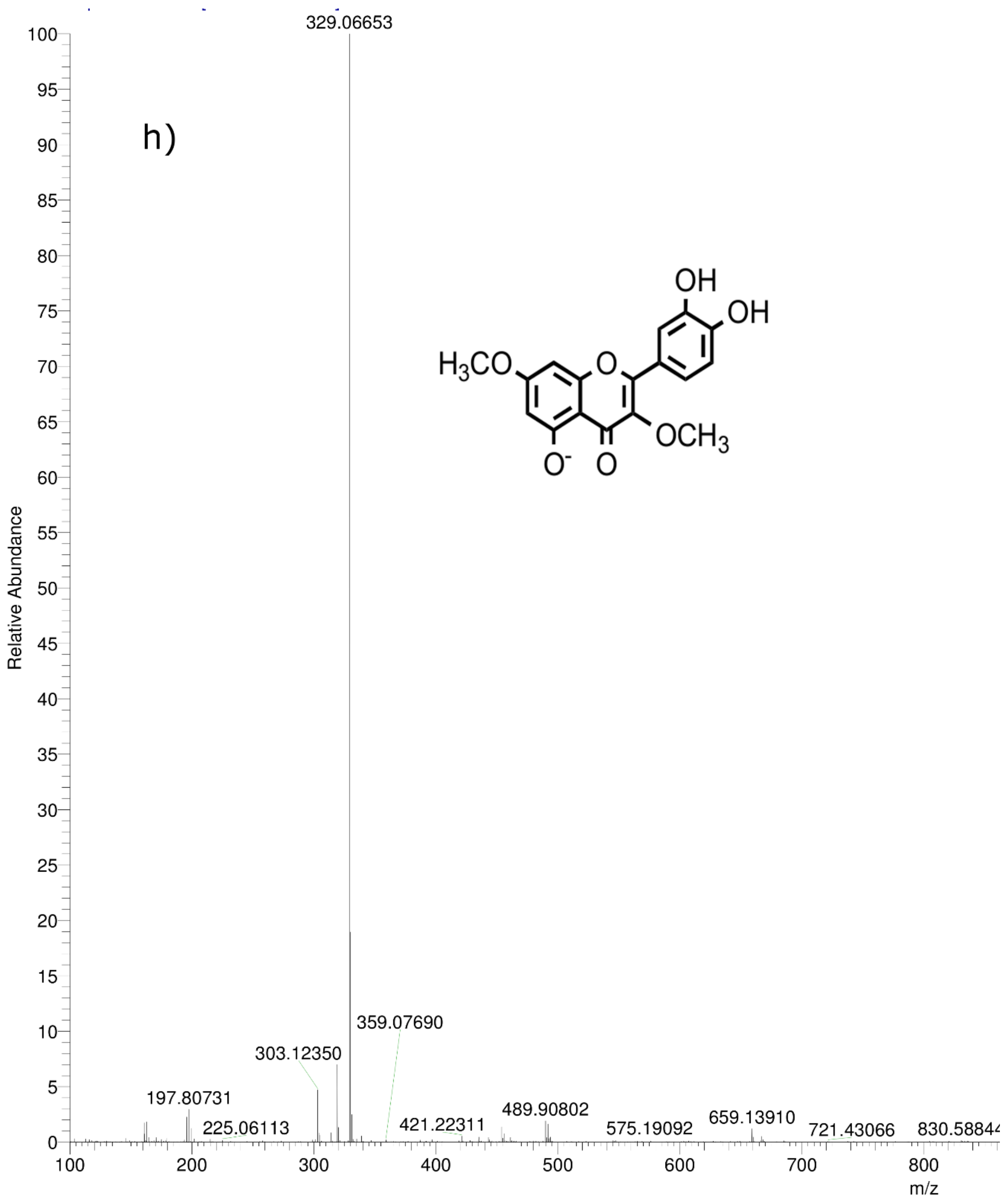

h)
329.06653
303.12350
359.07690
197.80731
225.06113
421.22311
489.90802
575.19092
659.13910
721.43066
830.5884
Relative Abundance
m/z
H3CO
OH
OH
O
OCH3
O-
O

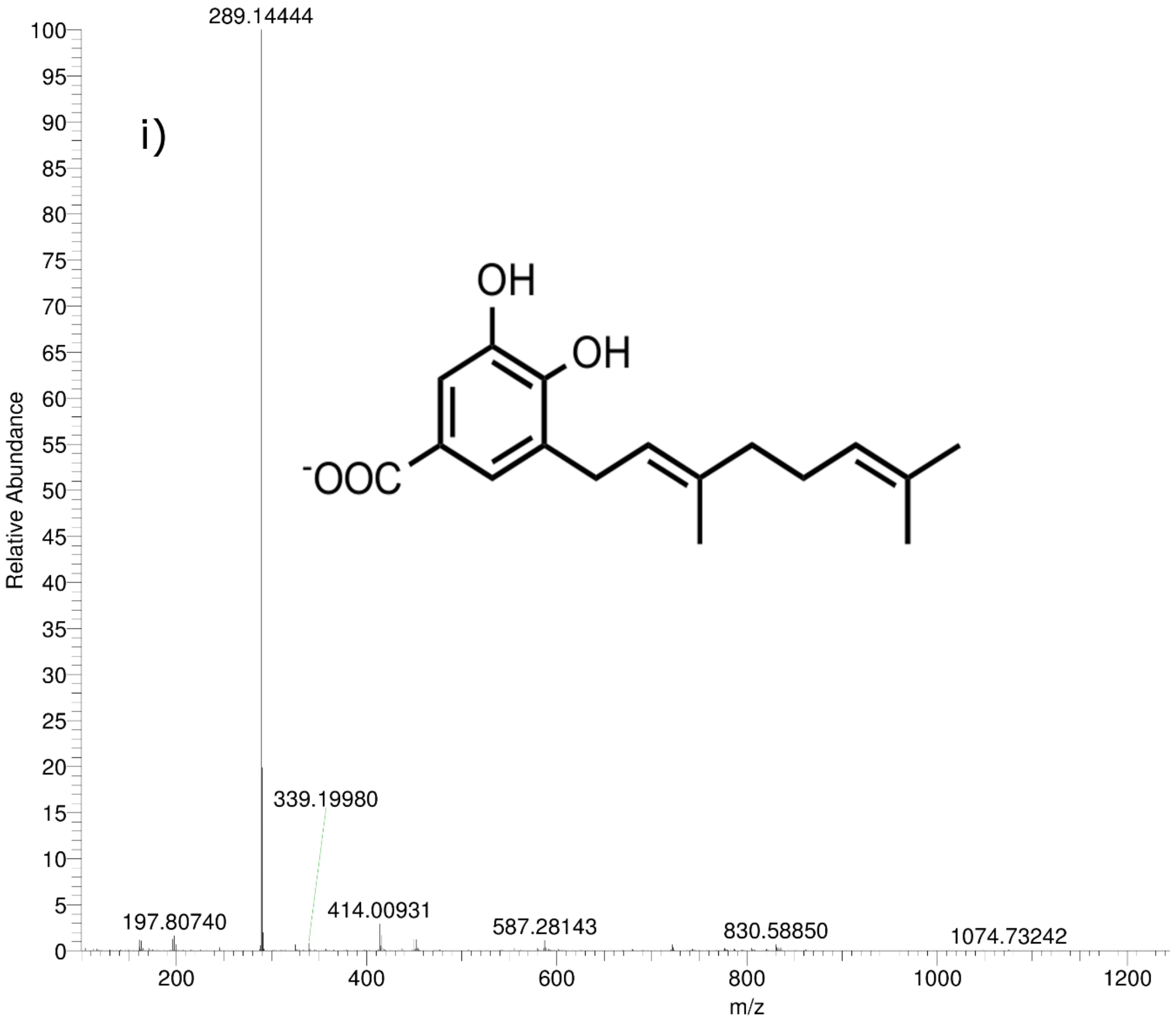

289.14444
i)
OH
OH
-OOC
339.19980
197.80740
414.00931
587.28143
830.58850
1074.73242
Relative Abundance
m/z

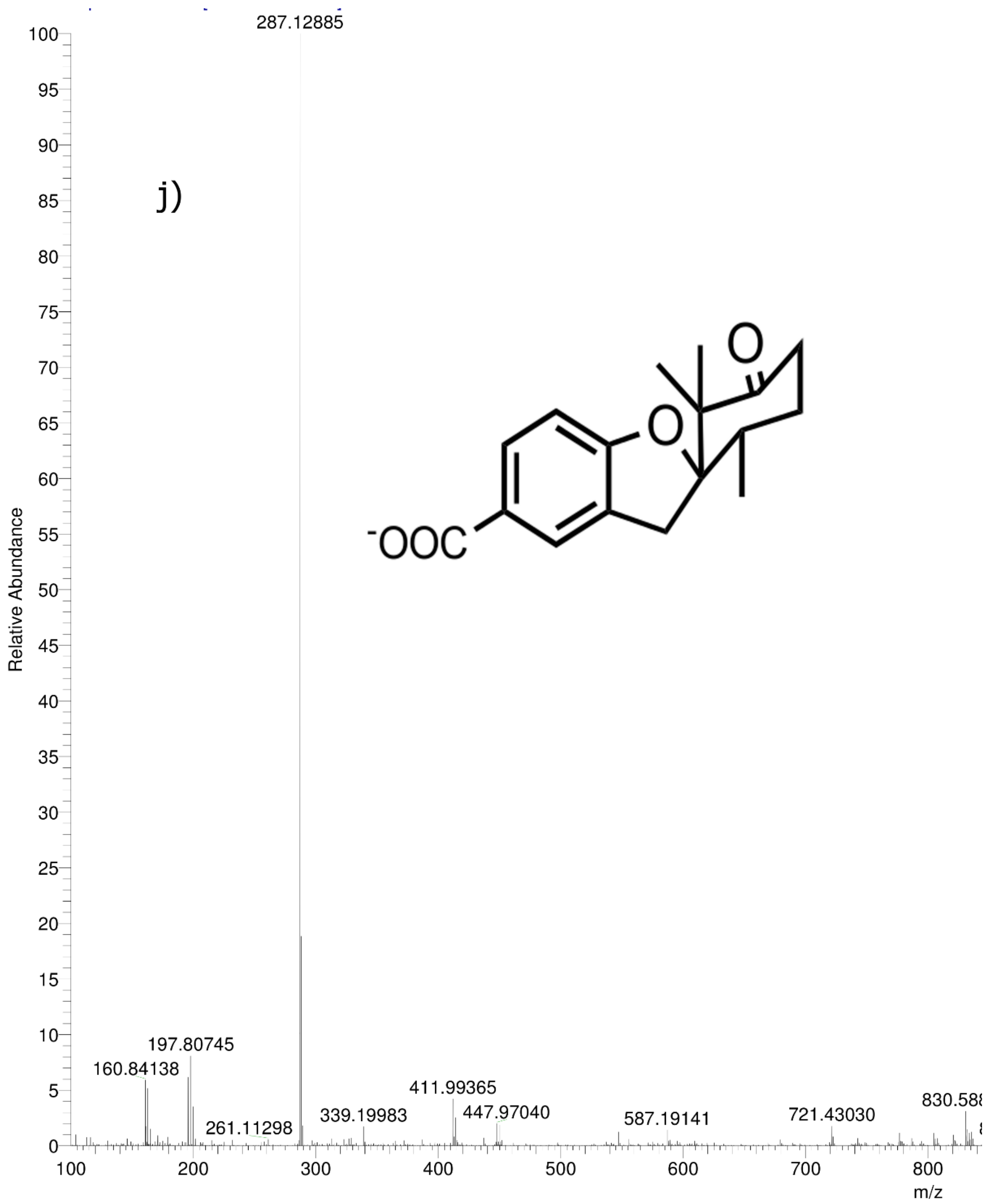

j)
287.12885
197.80745
160.84138
261.11298
339.19983
411.99365
447.97040
587.19141
721.43030
Relative Abundance
m/z
-OOC
O

Fig. S2.^{1}H NMR (300 Mhz) spectra for compound **1** peak 41 (pinostrobin) in $CDCl_3$ (J in Hz in parentheses).

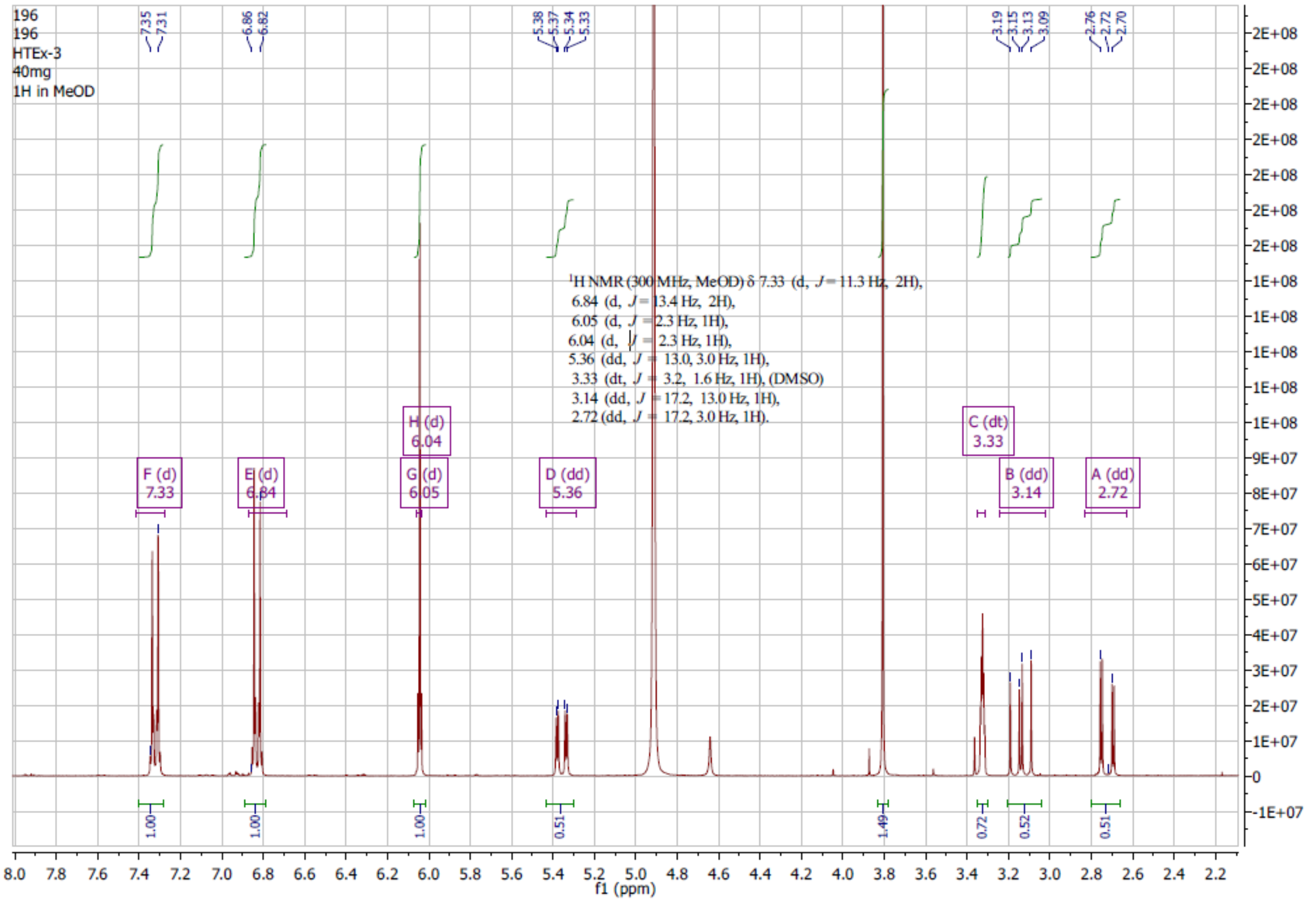

Fig. S3. Ampliation ^{1}H NMR spectra for compound 1, peak 41 (pinostrobin) in $CDCl_3$ (J in Hz in parentheses).

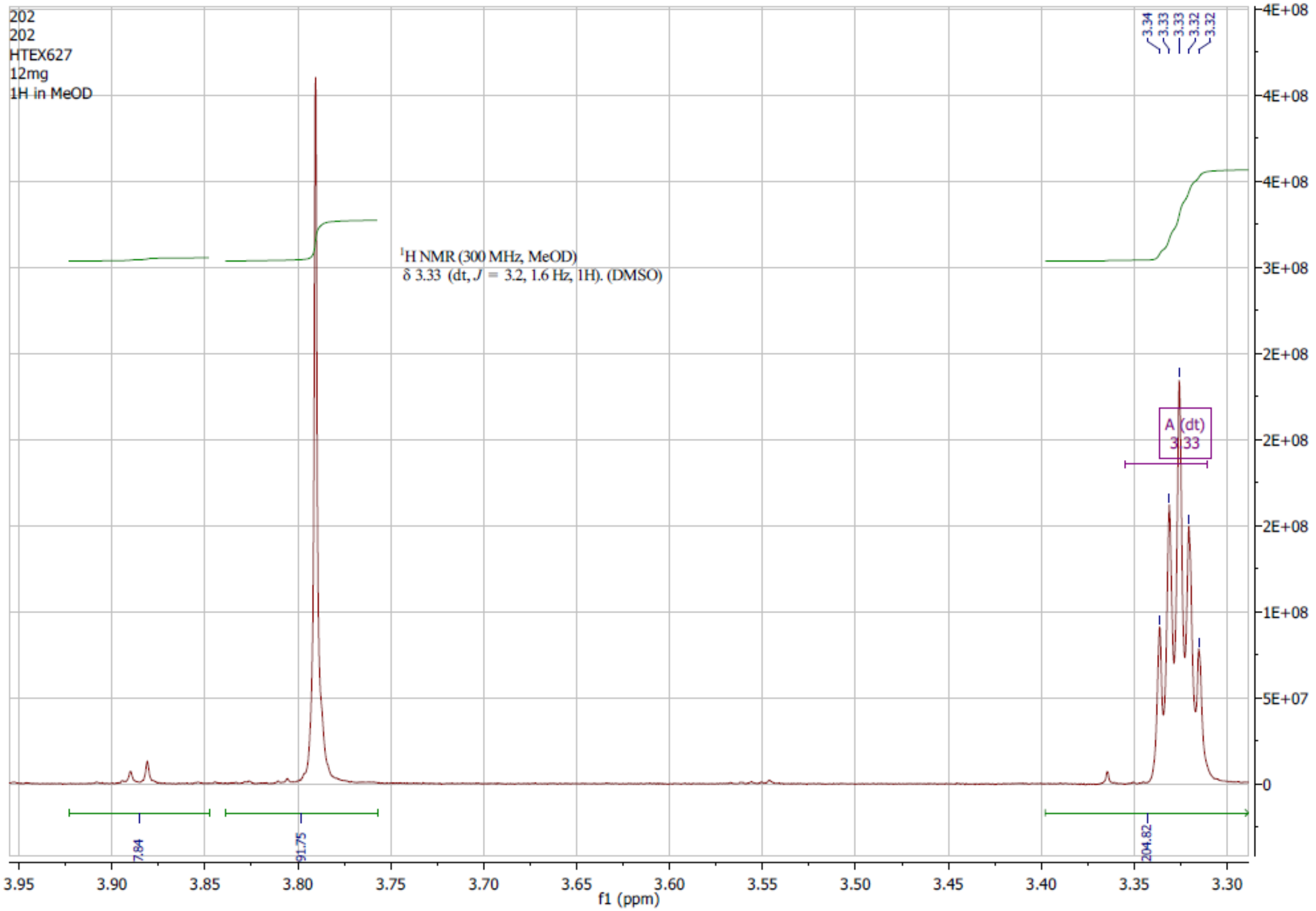

Fig. S4.^{13}C NMR (100.25 Mhz) spectra for compound 1, peak 41 (pinostrobin) in $CDCl_3$ (J in Hz in parentheses).

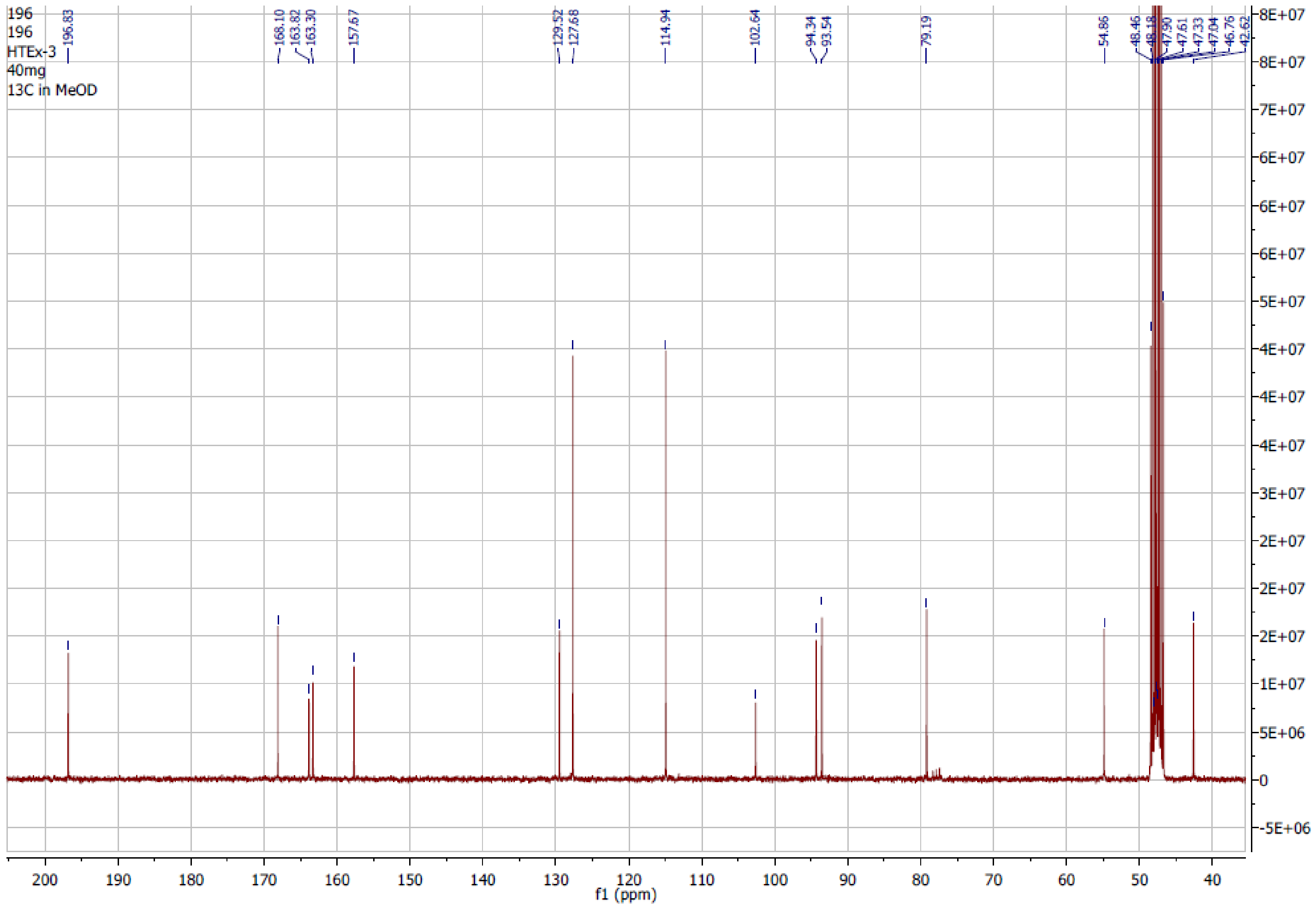

Fig. S5.^{1}H NMR (300 MHz) spectra for compound 2, peak 40 (pinocembrin) in $CDCl_3$ (J in Hz in parentheses).

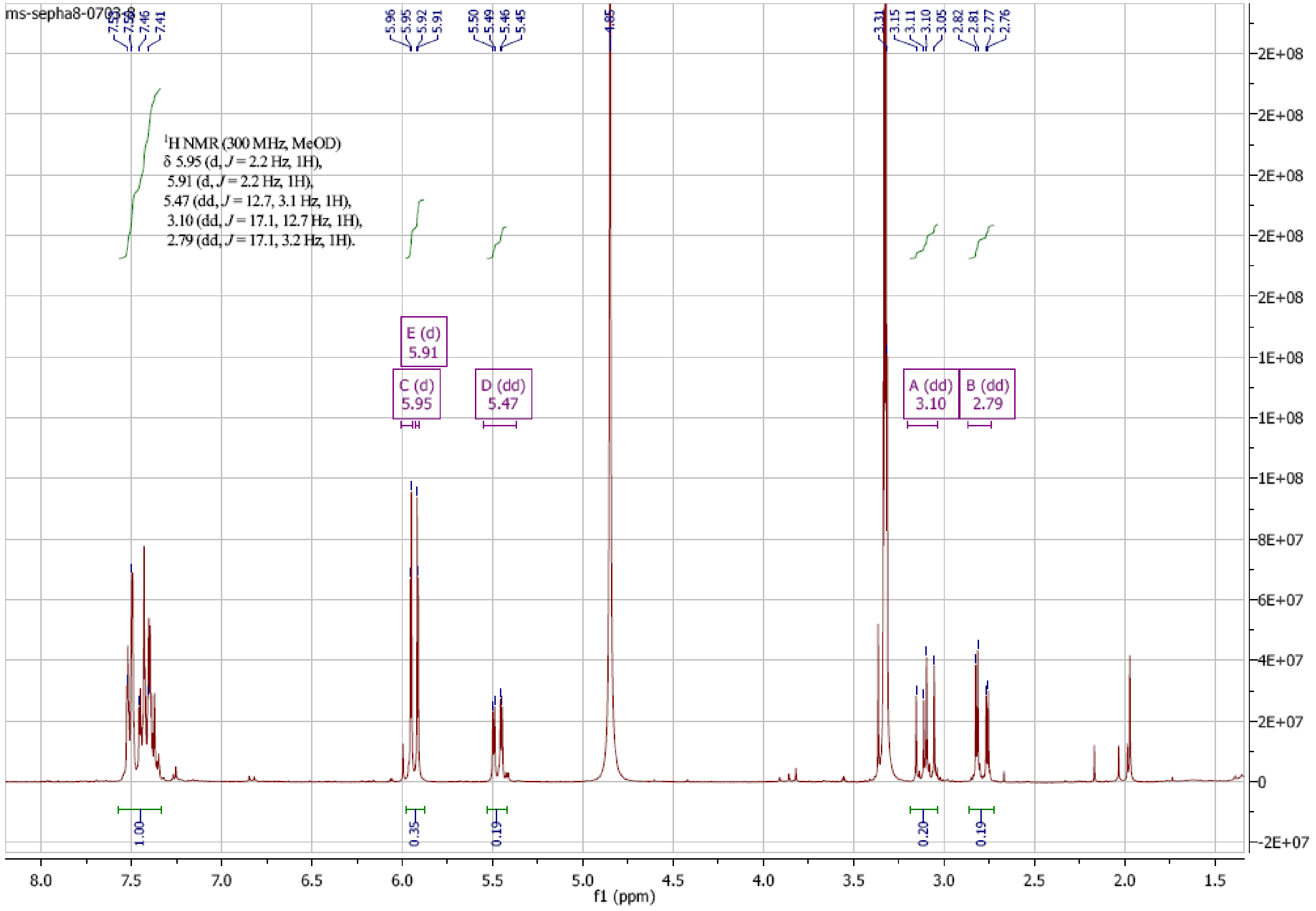

Fig. S6. DEPT 45 NMR (100.25 Mhz) spectra for compound 2, peak 40 (pinocembrin) in $CDCl_3$ (J in Hz in parentheses).

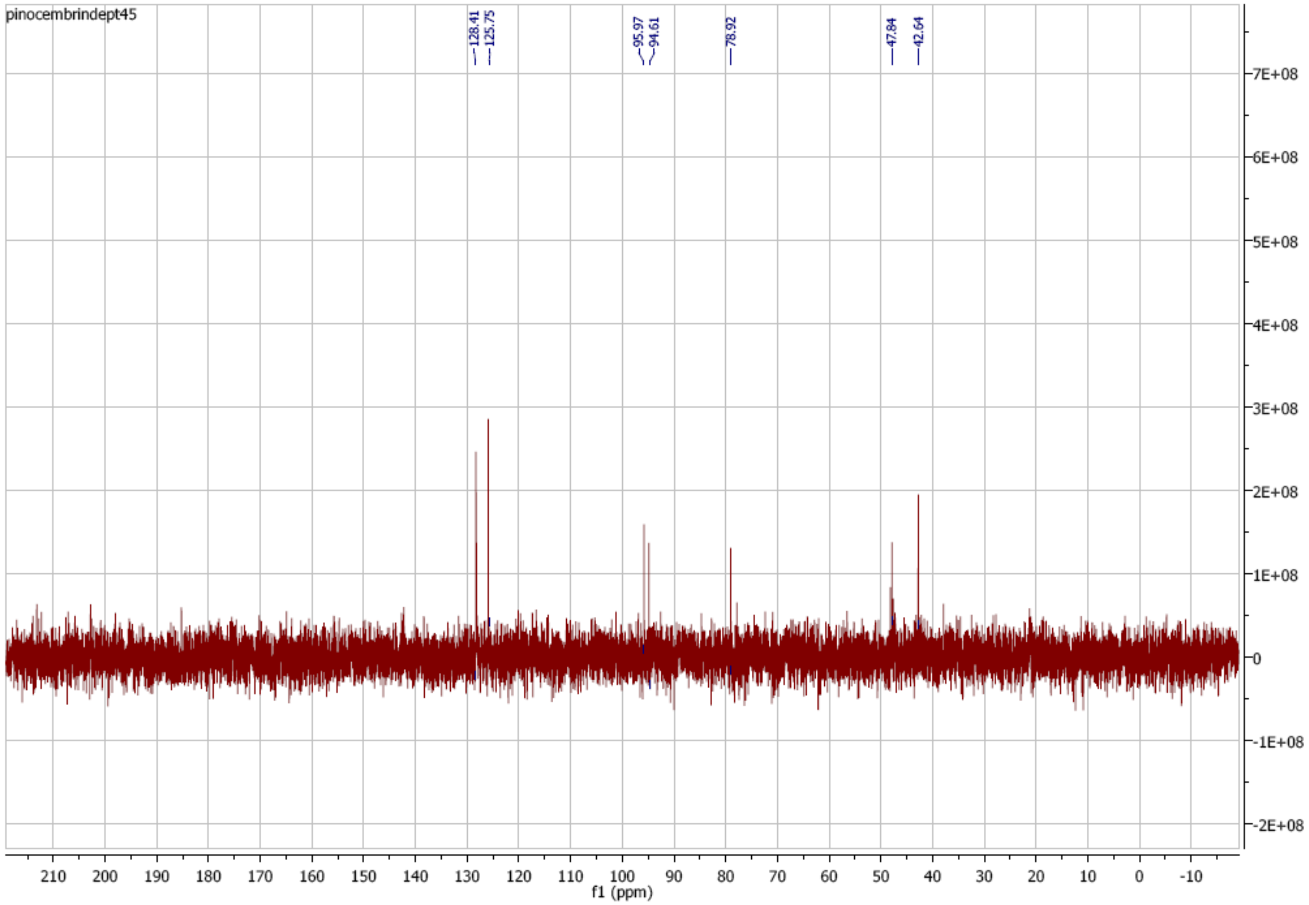

Fig. S7. DEPT 135 NMR (100.25 Mhz) spectra for compound 2, peak 40 (pinocembrin) in $CDCl_3$.

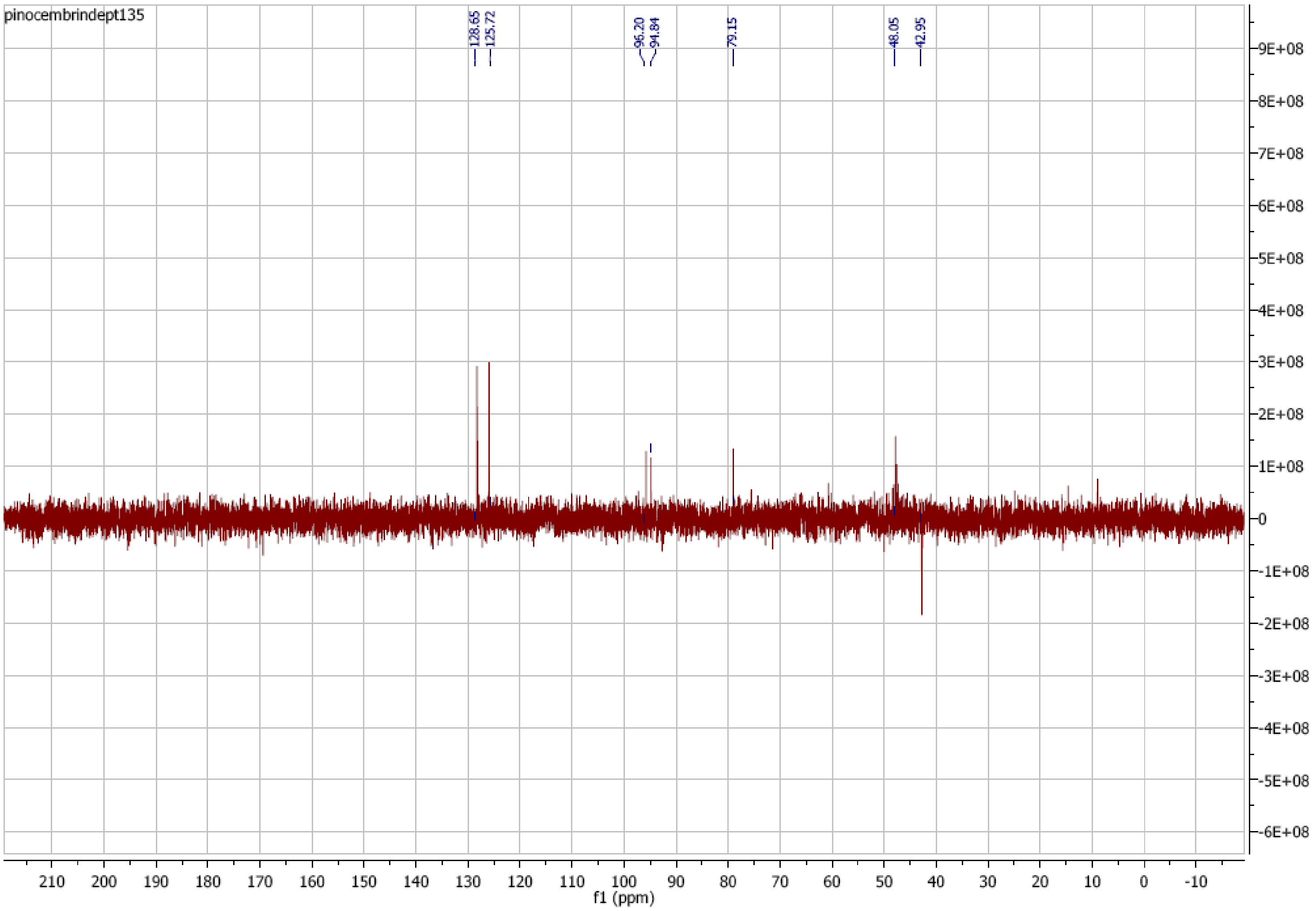

Fig. S8. ^{1}H NMR data (300 MHz) for compound 3, peak 32 (sakuranetin)

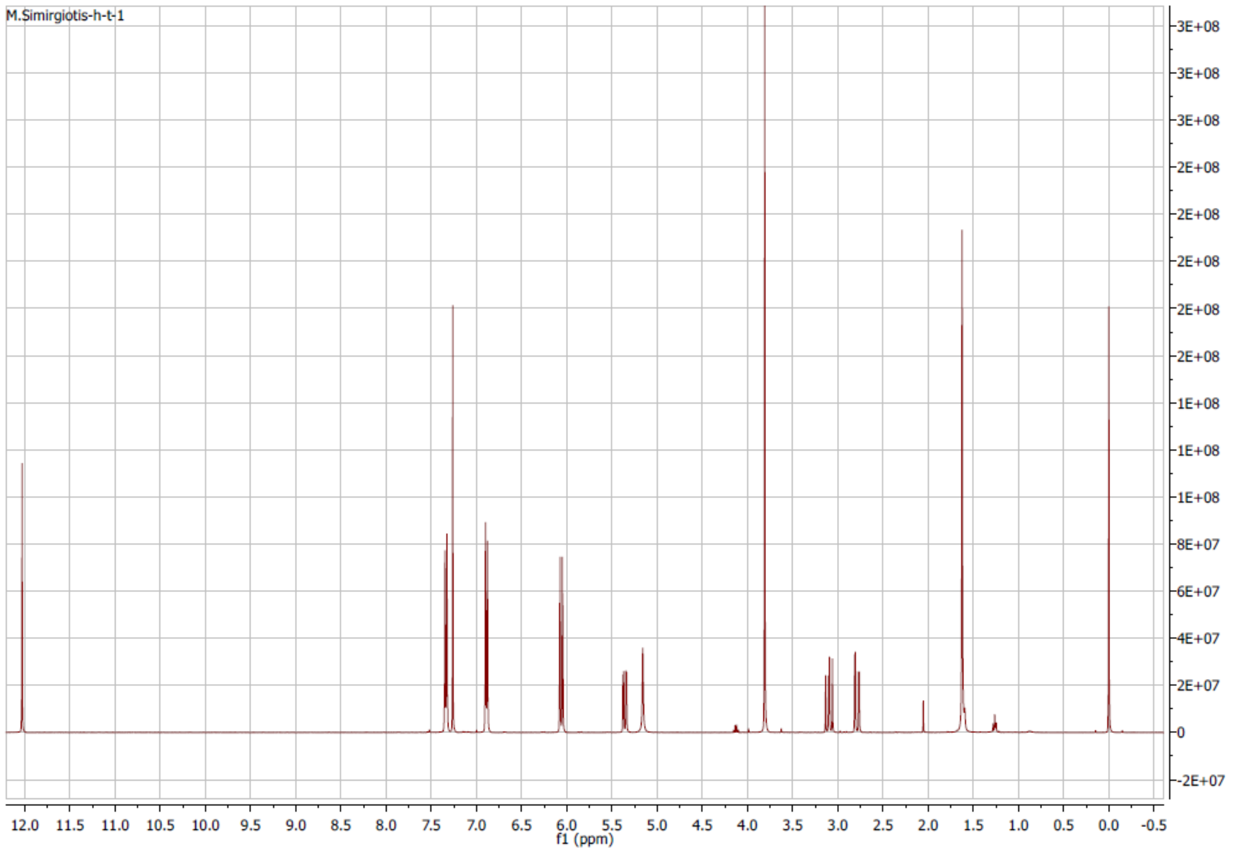

Fig. S9. [13] CNMR data (100 MHz) for compound 3, peak 32 (sakuranetin)

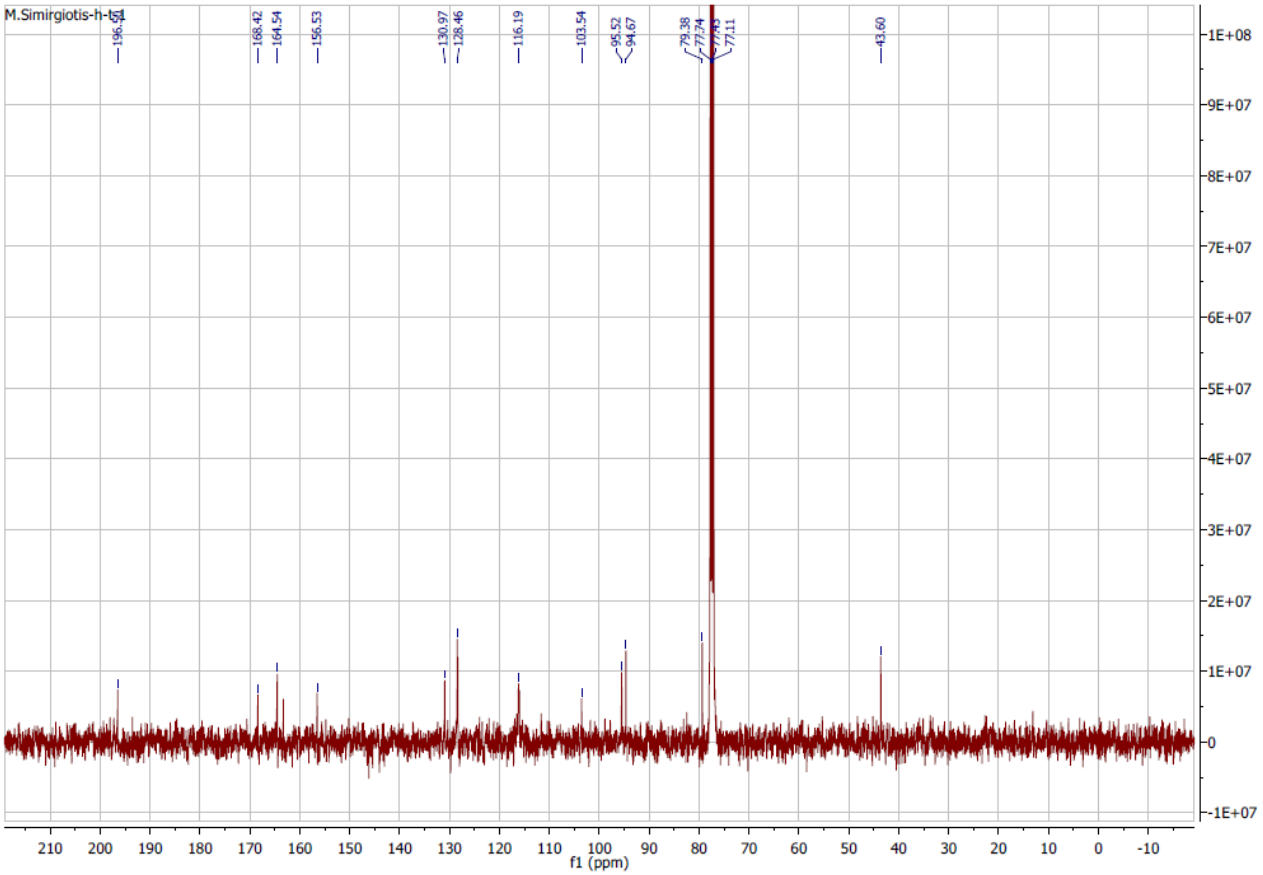

Fig. S10. DEPT 135 ^{13}C NMR data (100.25 MHz) for compound 3, peak 32 (sakuranetin)

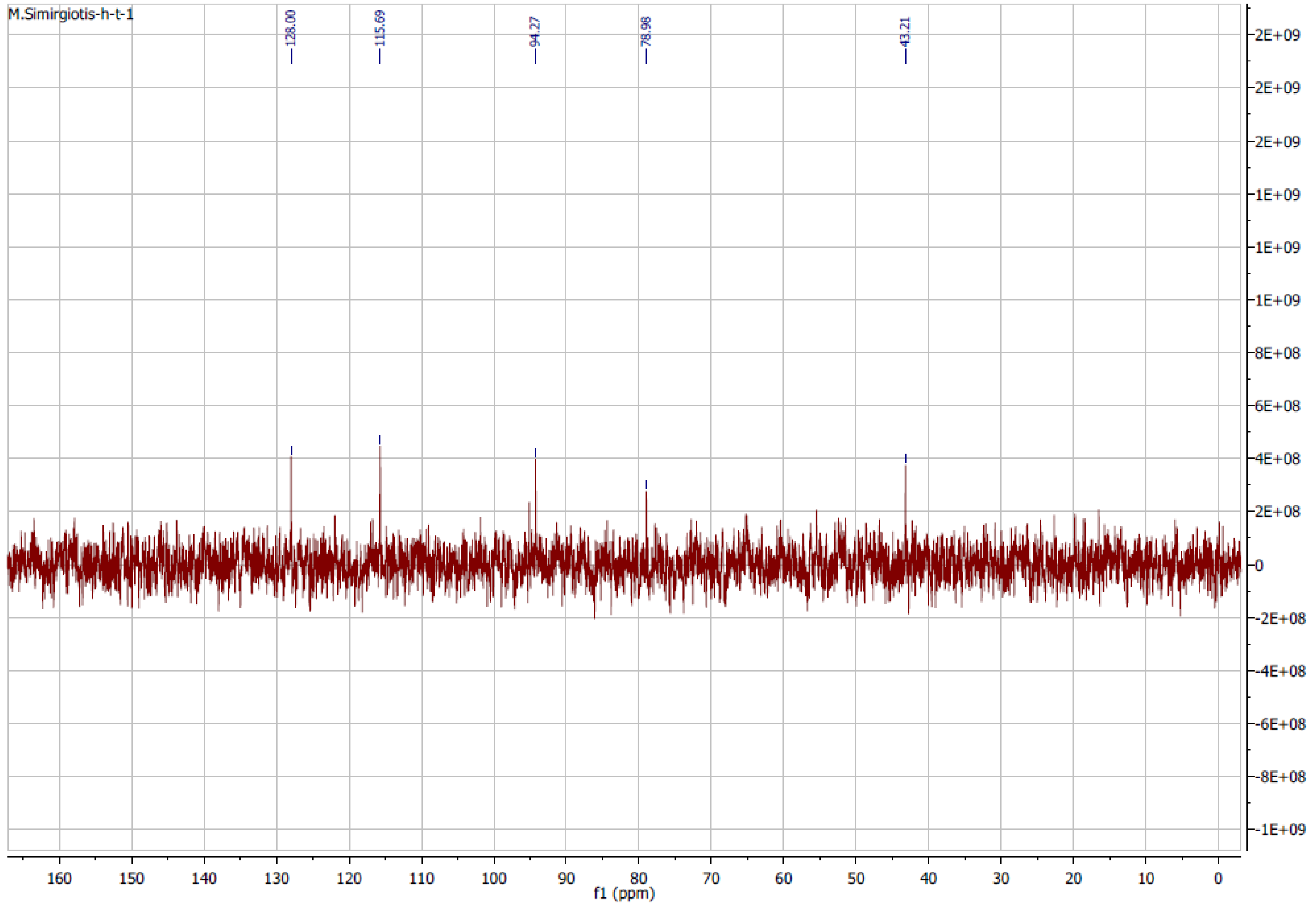

Fig. S11. COSY ^{1}HNMR data (300 MHz) for compound 3, peak 32 (sakuranetin)

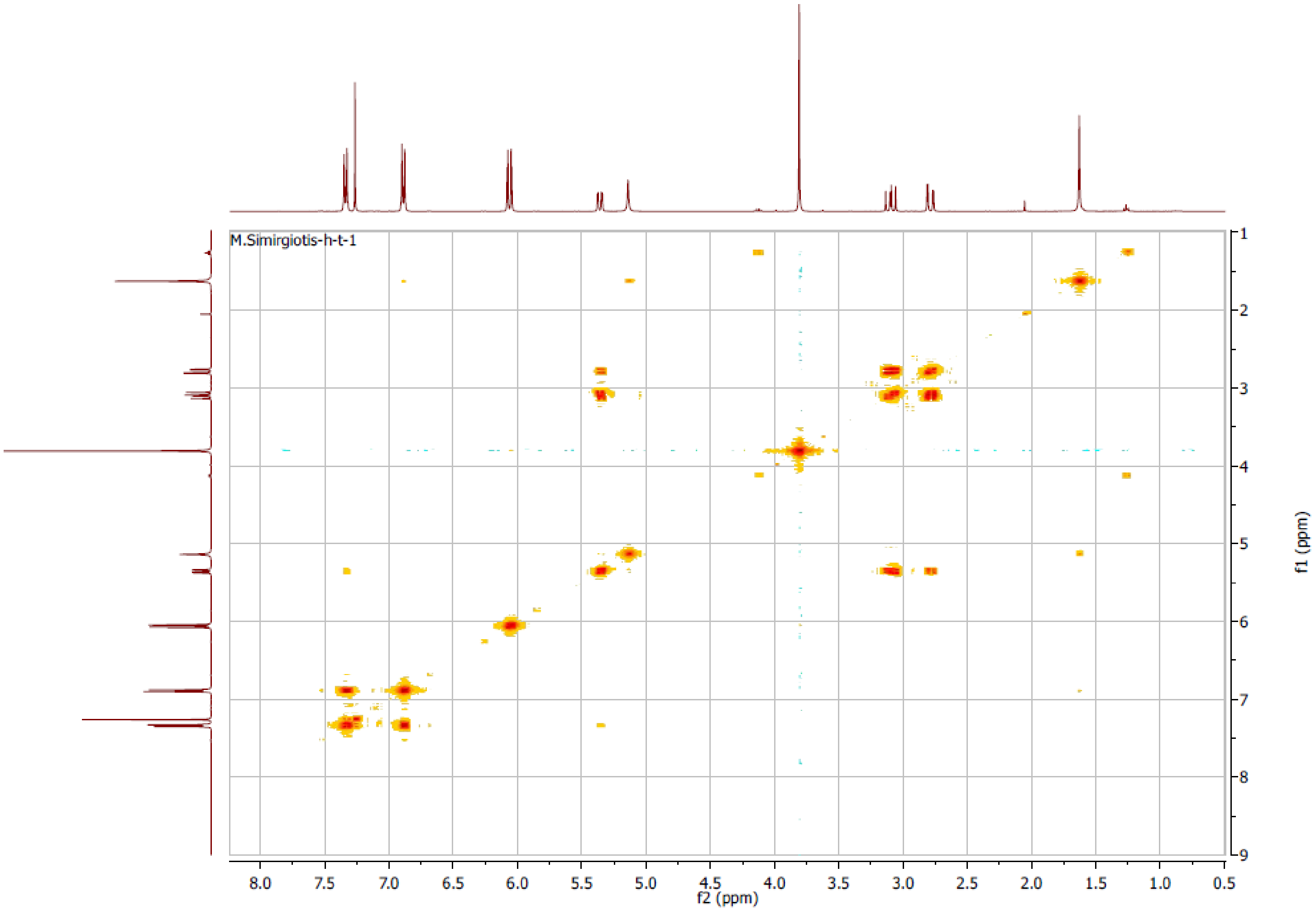

Fig. S12. HMQC ^{13}C NMR data (100.25 MHz) for compound 3, peak 32

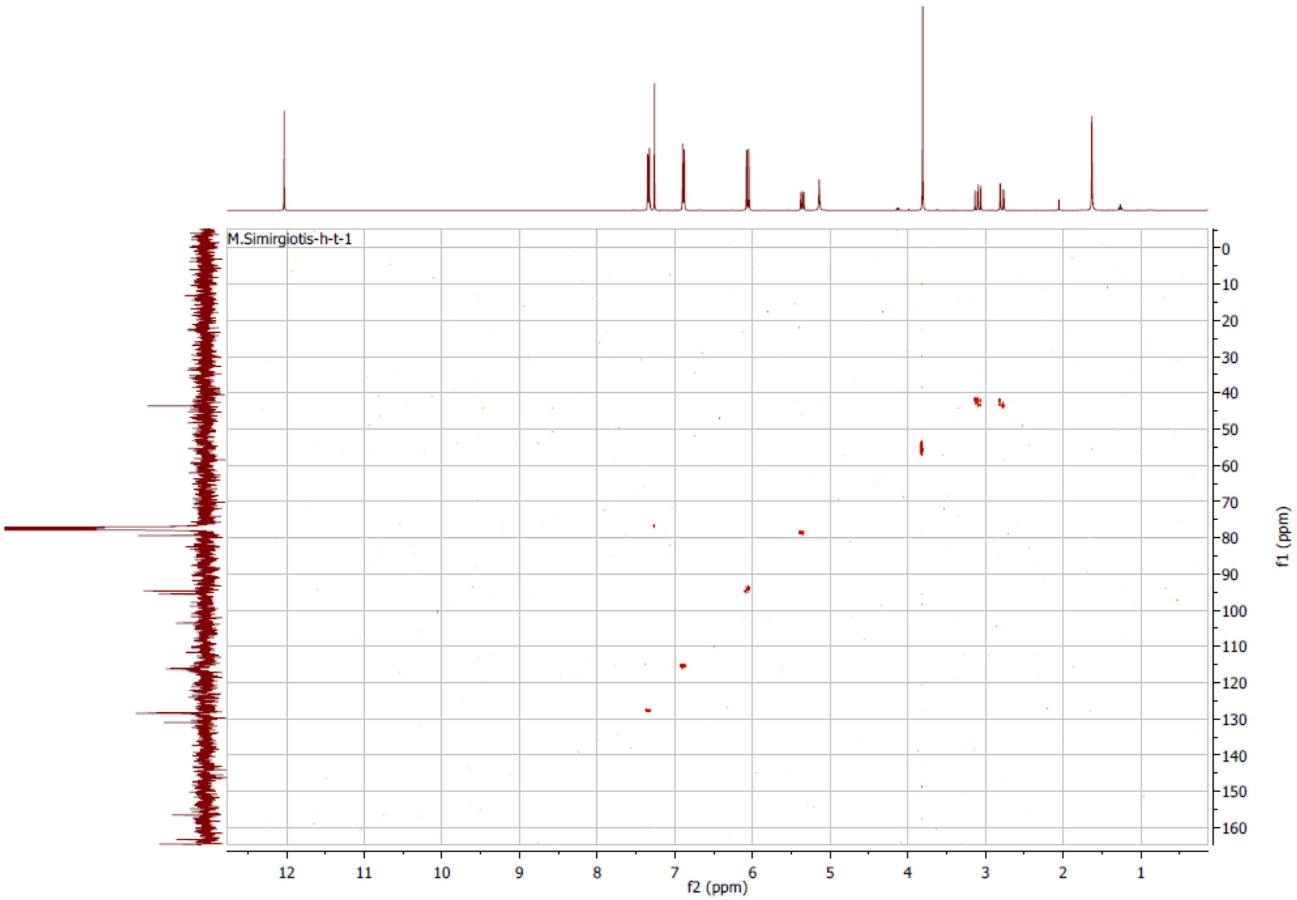

Fig. S13. HMBC ^{13}C NMR data (100.25 MHz) for compound 3, peak 32

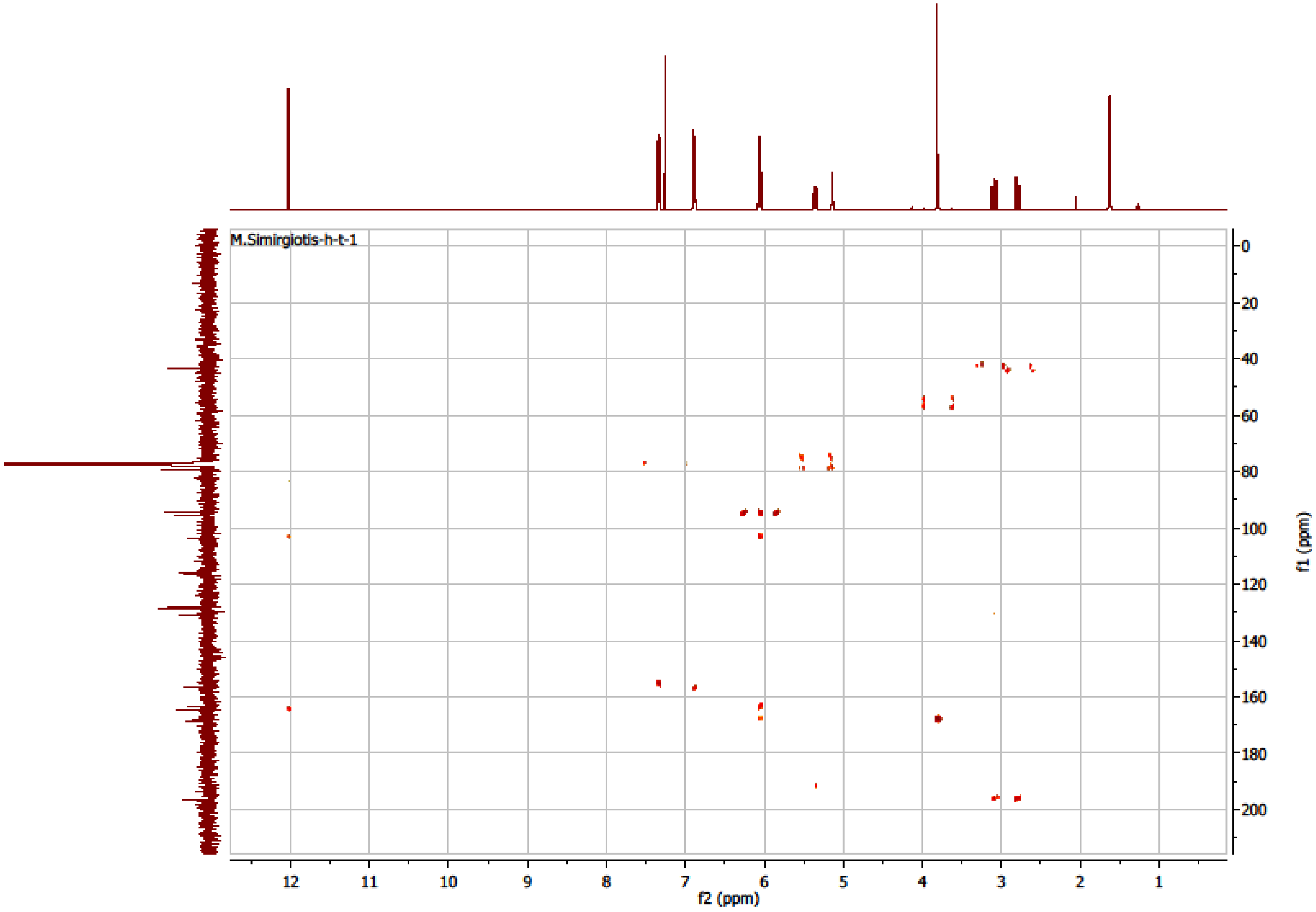

Fig. S14.^{1}H NMR (300 Mhz) spectra for compound 4, peak 37 (7-methoxykaepmferol) in $CDCl_3$ (J in Hz in parentheses).

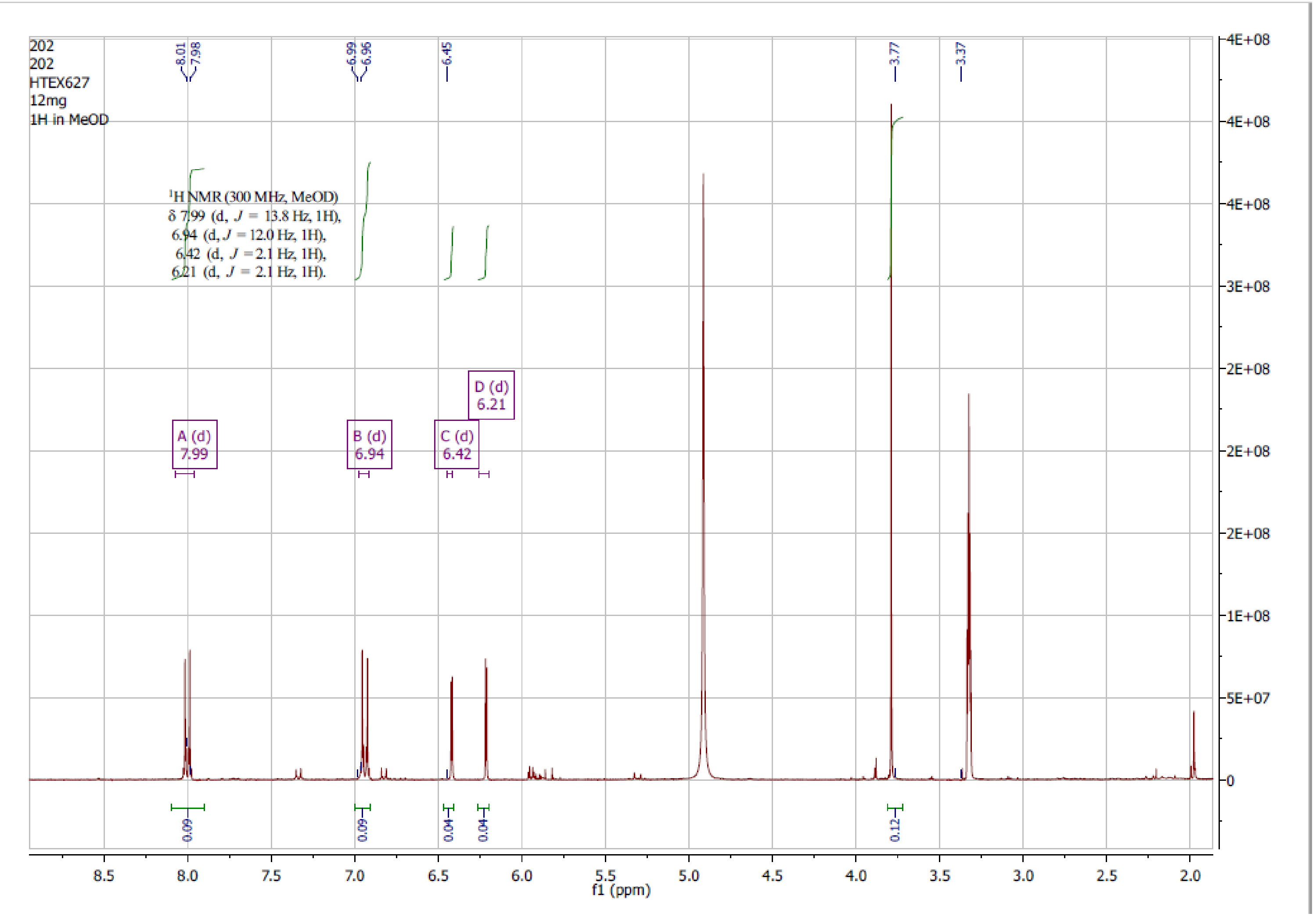

Fig. S15. Ampliation ^{1}H NMR spectra for compound 4, peak 37 (7-methoxykaepmferol) in $CDCl_3$ (J in Hz in parentheses).

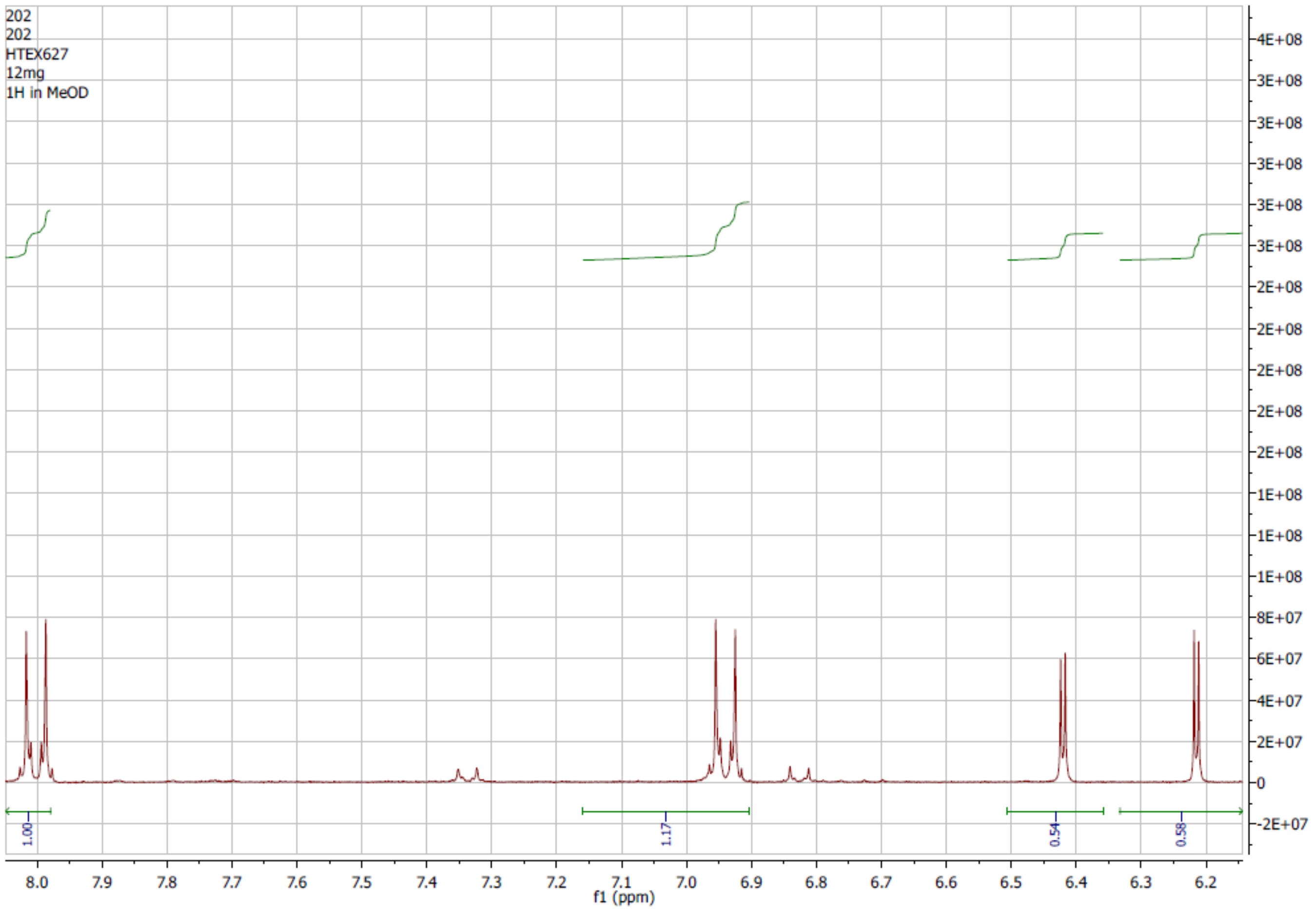

Fig. S16.^{13}C NMR (100.25 Mhz) spectra for compound 4, peak 37 (7- methoxykaepmferol) in $CDCl_3$ (J in Hz in parentheses).

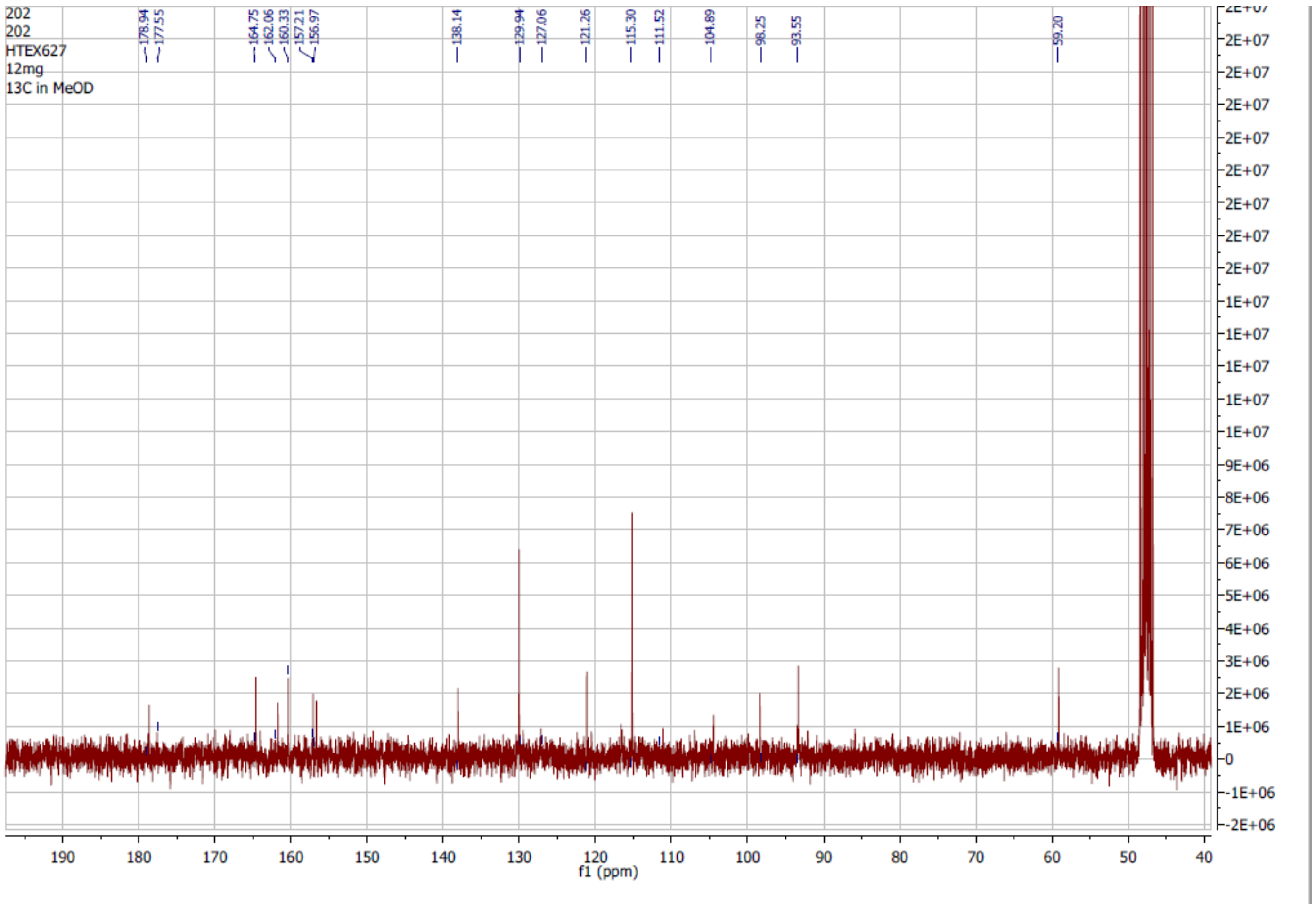

Fig. S17.^{1}H NMR (300 Mhz) spectra for compound 5, peak 52 (4,5-dihydroxy-3-geranyl-benzoic acid) in $CDCl_3$ (J in Hz in parentheses).

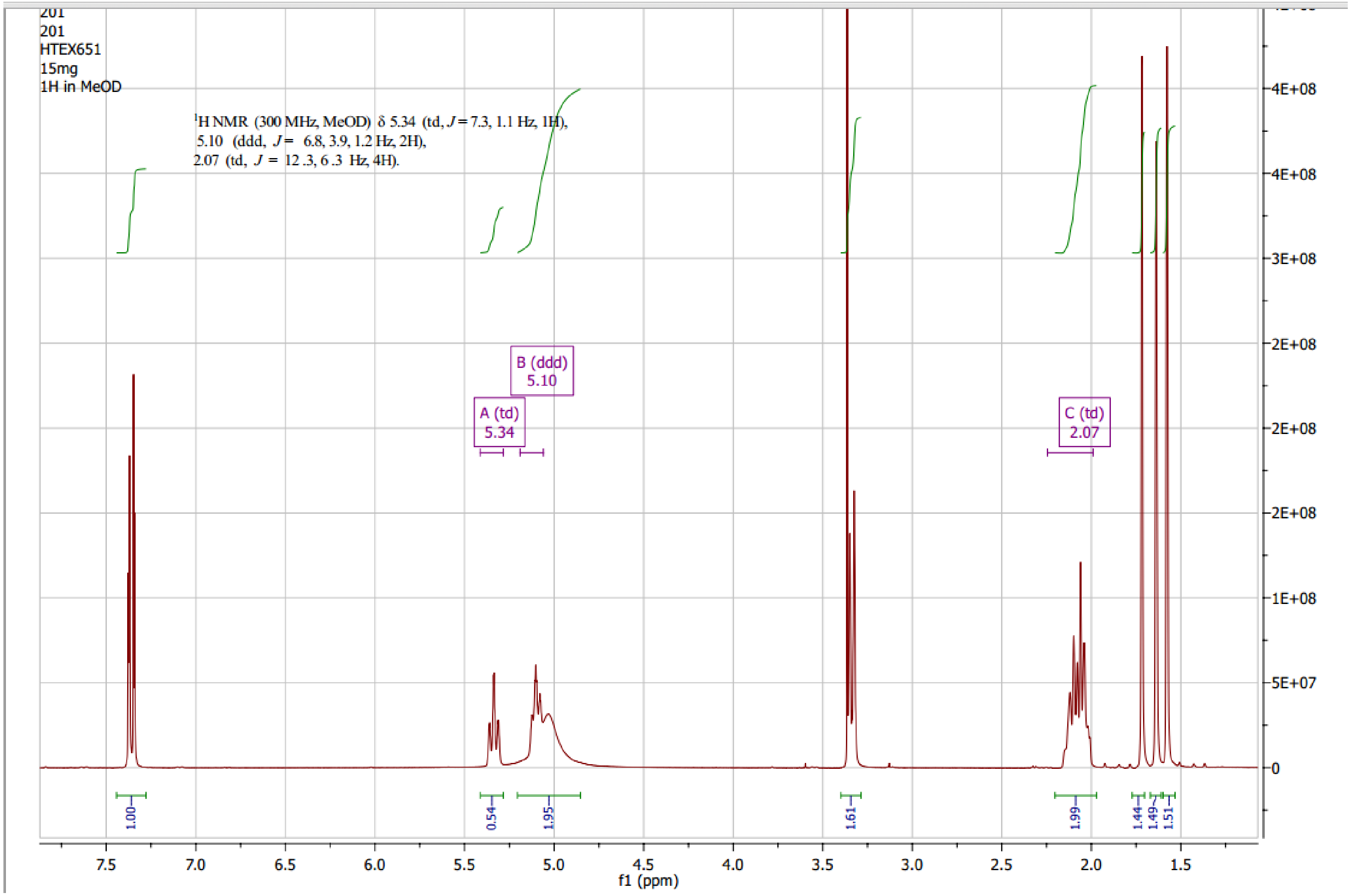

Fig. S18. ^{13}C NMR data (100.25 MHz) for compound 5, peak 52. (4,5-dihydroxy-3-geranyl-benzoic acid)

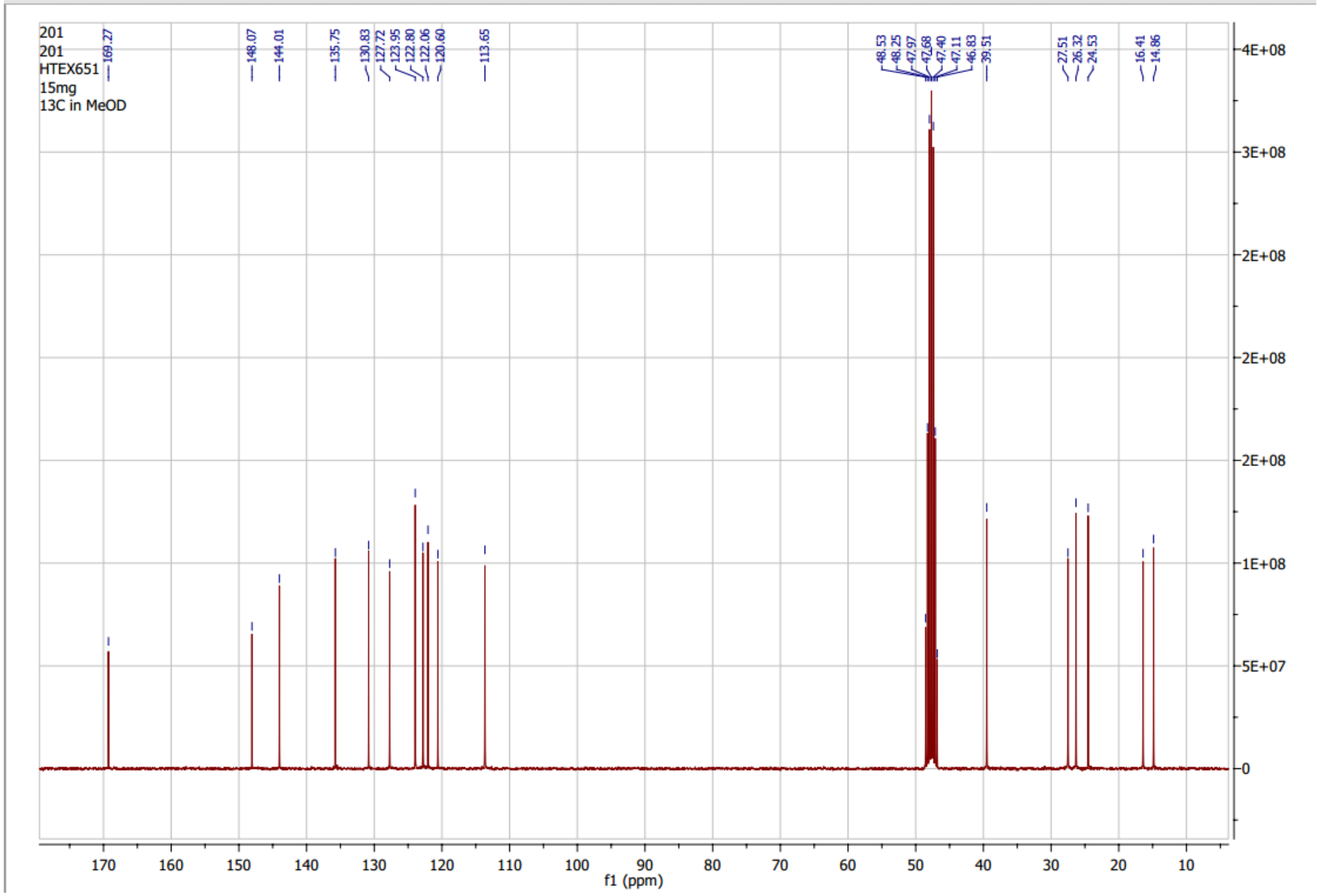

Fig. S19. DEPT 135 ^{13}C NMR data (100.25 MHz) for compound 5, peak 52. (4,5-dihydroxy-3-geranyl-benzoic acid)

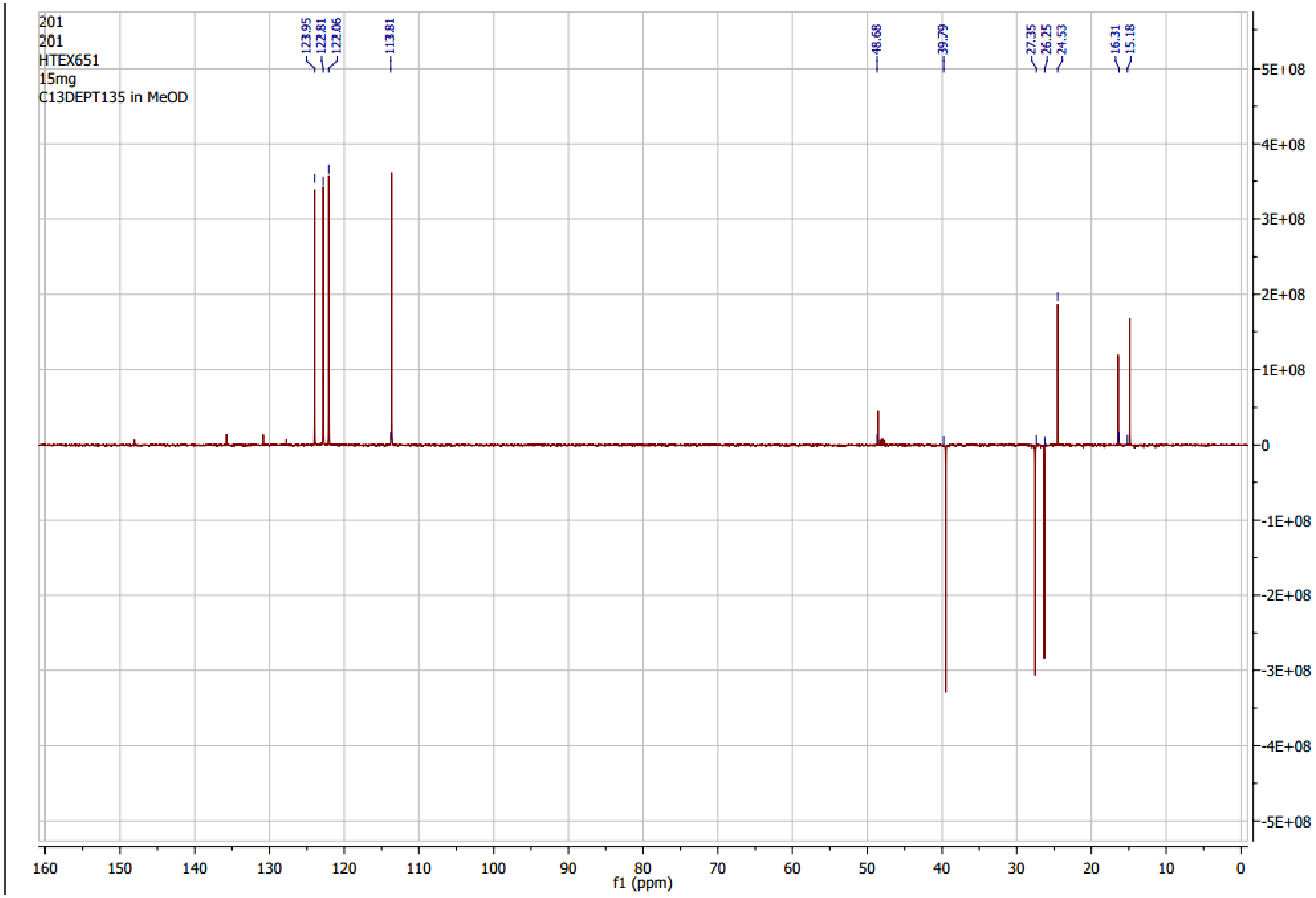

Fig. S20. COSY ^{1}HNMR data (300 MHz) for compound 5, peak 52 (4,5-dihydroxy-3-geranyl-benzoic acid)

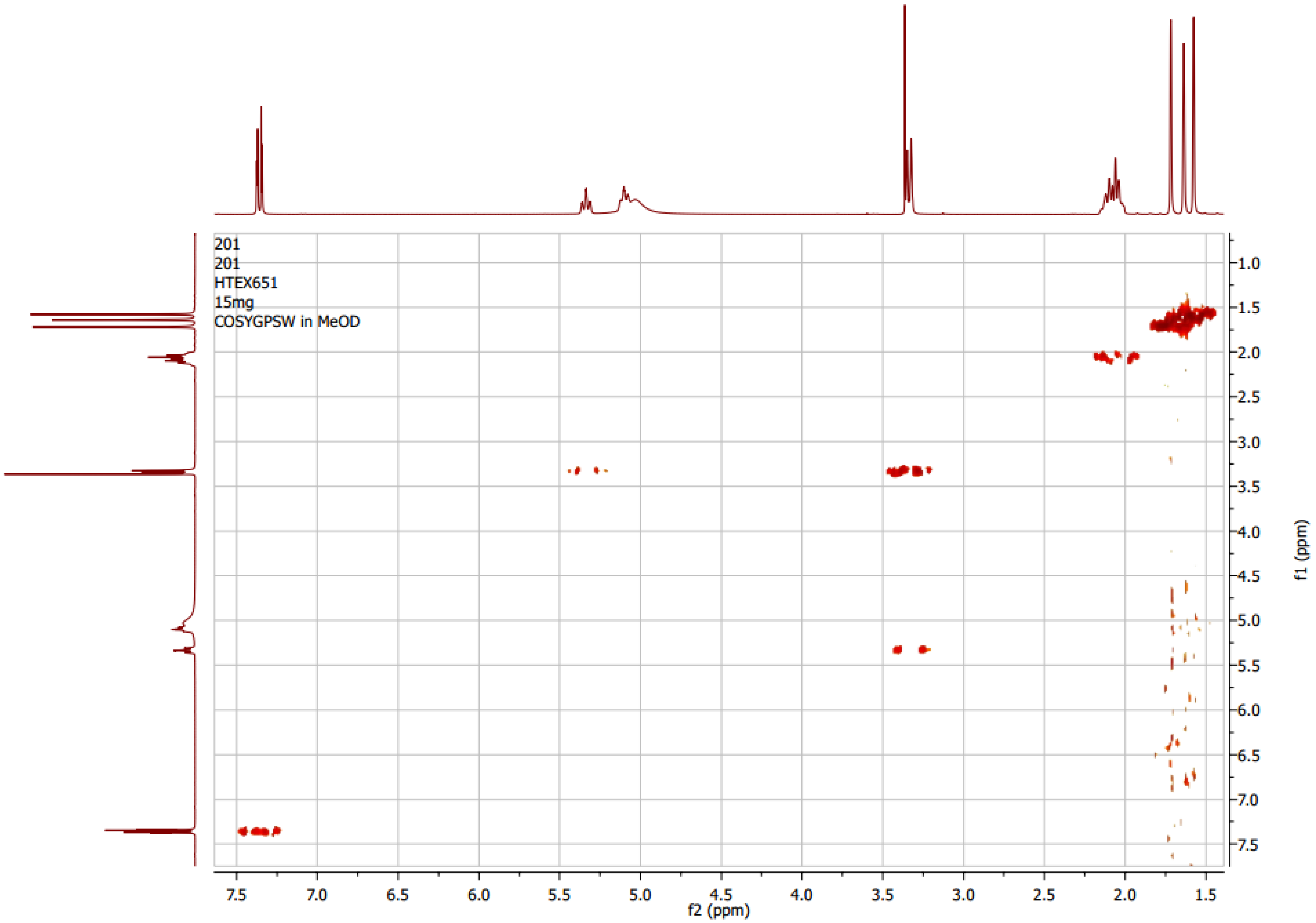

Fig. S21. HSQC ^{13}C NMR data (100.25 MHz) for compound 5, peak 52. (4,5-dihydroxy-3-geranyl-benzoic acid)

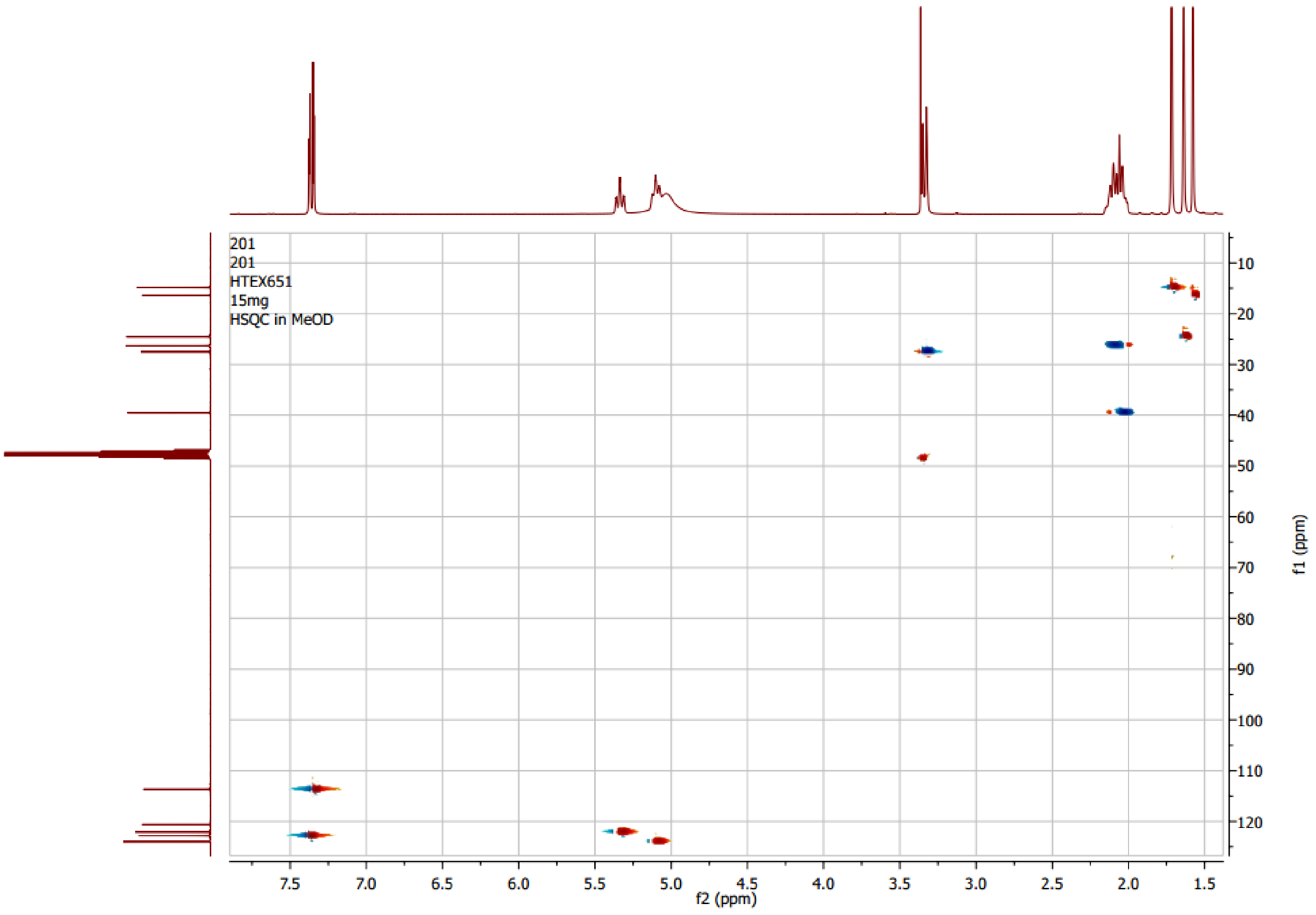

Fig. S22. HMBC ^{13}C NMR data (100.25 MHz) for compound 5, peak 52. (4,5-dihydroxy-3-geranyl-benzoic acid)

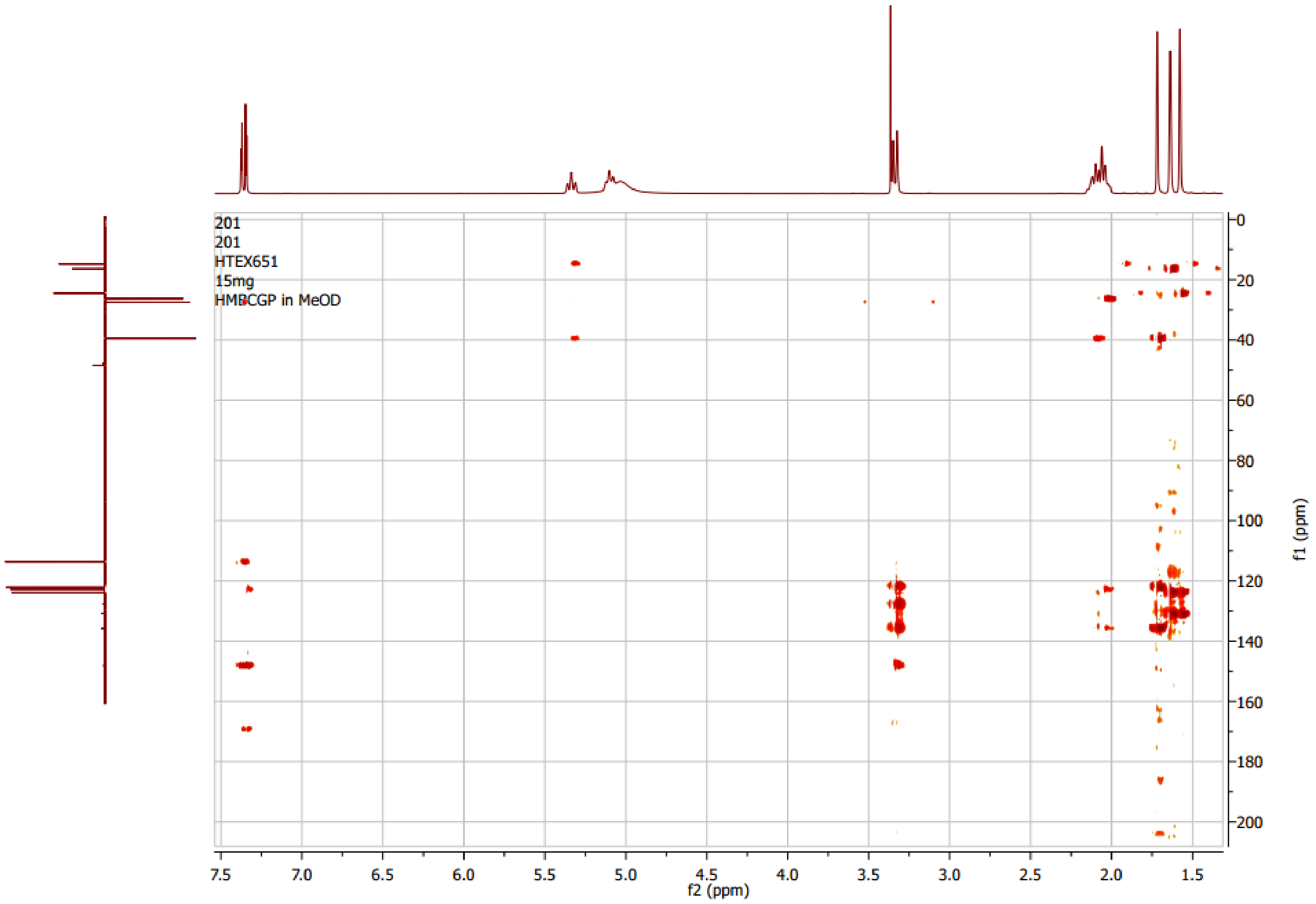

Fig. S23: Uv spectra of compound 5, peak 52 (4,5-dihydroxy-3-geranyl-benzoic acid)

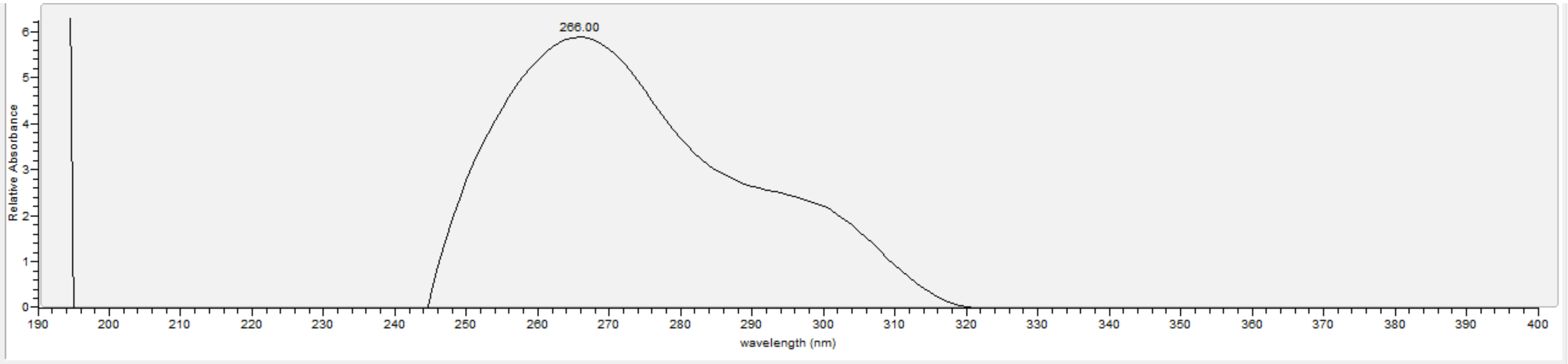

Methods

1. *Phenolic content (TPC) estimation.*

The TPC method was based on the Folin–Ciocalteu method. A total of 20 μL of the diluted extract (500 μg/mL) were mixed with 100 μL of 10% (vol/vol) of Folin–Ciocalteu reagent and shaken. After 5 mins, 75 μL of $NaCO_3$ (700 mM) was added, and absorbance measured at 765 nm using a microplate reader after 1 hour at room temperature. Gallic acid dilutions (0-1000 μg/mL) were used as standards for calibration. Data from these multiple experiments were presented as milligram of gallic acid equivalent per gram of dry extract.

2. *Total flavonoid content (TFC) estimation.*

Briefly, a mixture of 50 μL extracts, 25 μL of aluminum chloride (10%), 80 μL of methanol, and 25 μL of 1 M potassium acetate were placed in a micro-plate, and absorbance read at 510 nm after incubation for 30 min. Analyses were carried out in quadruplicate and the results were expressed as mg quercetin equivalent per gram of dry extract.

3. *DPPH Radical Scavenging Activity Assay*

Briefly, a 0.2 mM solution of 1,1-diphenyl-2-picrylhydrazyl (DPPH) in methanol was prepared, and 70 μL of this solution was added to 20 μL of extract (0-1000 μg/mL). Trolox in concentrations of 0-1000 μg/mL was used as a standard reference antioxidant. Discoloration of the reaction mixture was measured at 517 nm after incubation for 30 min. The results were expressed as IC_{50} (concentration of extract or standard in μg/mL required to inhibit 50% of DPPH radical present in solution). Analyses were carried out in quadruplicate.

4. *Determination of ABTS radical-scavenging activity*

Briefly, a mixture of 2.5 mM $K_2S_2O_8$, methanol and ABTS in phosphate buffer saline at pH 7.4 was corrected for absorbance at 734 nm, and kept in the dark at 22°C. A mixture of 180 μL of ABTS in PBS and 20 μL of PBS was used as the blank solution. The radical scavenging properties were measured using Trolox as a standard, calculated as concentration required to scavenge 50% of ABTS radicals, and expressed as IC_{50} (μg/mL). Experiments were performed in multiples of two

5. *Ferric reducing antioxidant power (FRAP) assay.*

The FRAP reagent composed of 300 mM buffer acetate at pH 3.6, 2,4,6-tris-(2-pyridyl)-s-triazine 10 mM (TPTZ) in hydrochloric acid 40 mM and $FeCl_3.6H_2O$ aqueous solution 20 mM in the ratio of 10:1:1 (v/v). 70 μL FRAP solution was mixed with 10 μL extract solution at 500 μg/mL, absorbance was read at 593 nm, and compared with 0 - 500 μg/m trolox solution (standard). Results were presented as mg of trolox/g of dry extract. Experiments were performed in multiples of two.
